# Robust inverse probability weighted estimators for doubly truncated Cox regression with closed-form standard errors

**DOI:** 10.1007/s10985-025-09650-5

**Published:** 2025-04-15

**Authors:** Omar Vazquez, Sharon X. Xie

**Affiliations:** Department of Biostatistics, Epidemiology and Informatics, University of Pennsylvania, Philadelphia, PA USA

**Keywords:** AIDS, Cox model, double truncation, robust inference, 62N02, 62N03

## Abstract

Survival data is doubly truncated when only participants who experience an event during a random interval are included in the sample. Existing methods typically correct for double truncation bias in Cox regression through inverse probability weighting via the nonparametric maximum likelihood estimate (NPMLE) of the selection probabilities. This approach relies on two key assumptions, quasi-independent truncation and positivity of the sampling probabilities, yet there are no methods available to thoroughly assess these assumptions in the regression context. Furthermore, these estimators can be particularly sensitive to extreme event times. Finally, current double truncation methods rely on bootstrapping for variance estimation. Aside from the unnecessary computational burden, there are often identifiability issues with the NPMLE during bootstrap resampling. To address these limitations of current methods, we propose a class of robust Cox regression coefficient estimators with time-varying inverse probability weights and extend these estimators to conduct sensitivity analysis regarding possible non-positivity of the sampling probabilities. Also, we develop a nonparametric test and graphical diagnostic for verifying the quasi-independent truncation assumption. Finally, we provide closed-form standard errors for the NPMLE as well as for the proposed estimators. The proposed estimators are evaluated through extensive simulations and illustrated using an AIDS study.

**Supplementary Information:**

The online version contains supplementary material available at 10.1007/s10985-025-09650-5.

## Introduction

Survival data is doubly truncated when inclusion in the sample requires that the event of interest occurs between two random truncation times, termed the study entry and end times. For example, autopsy-confirmation remains the gold standard for verifying Alzheimer’s disease (AD) status. If only autopsy-confirmed cases are included in an analysis where the time origin is the time of AD symptom onset, however, the sample becomes biased due to excluding all patients that either succumb to the disease prior to enrollment or survive past the end of the study. Another example of double truncation is the AIDS data analyzed in Sect. 5. In this dataset, the inclusion criteria are that the event of interest, AIDS diagnosis, occurred after the discovery of the disease and before the end of the study, with time measured in months since HIV infection. Double truncation also occurs in non-biomedical applications (Dörre and Emura 2019). In astronomy, for example, Efron and Petrosian (1999) describe a sample of quasar data which consists of quasars with luminosity (the “event time" here) large enough to yield reliable redshifts, but small enough to avoid confusion with other stellar objects. Standard survival analysis methods for left truncated and right censored data cannot be directly applied to doubly truncated data, since the resulting estimates will generally be biased and classical martingale-based results no longer apply.

### Problem statement

A commonly used approach to account for double truncation bias in Cox regression is inverse probability weighting (Rennert and Xie 2018; Mandel et al. 2018), where the weights can be obtained by a nonparametric maximum likelihood estimator (NPMLE) under some conditions which will be described shortly (Efron and Petrosian 1999; Shen 2010; de Uña-Álvarez and Keilegom 2021). These inverse probability weighted estimators are popular because they are flexible, since they lack any modeling assumptions for the truncation distribution and can be easily computed using standard software. In particular, they can directly use some residual diagnostics from standard Cox regression software, unlike the conditional maximum likelihood estimator of Rennert and Xie (2022). Unfortunately, they currently suffer from several limitations that are specific to doubly truncated data.

First, in addition to the usual Cox model assumptions, these estimators rely on two key conditions. The first key condition is quasi-independence (see Sect. 3) between the truncation times and the event times and covariates. While tests for quasi-independence have been studied in the context of nonparametric analyses (Martin and Betensky 2005; Shen 2011), there are no available methods to thoroughly assess this assumption under double truncation when covariates are involved. Under one-sided truncation, quasi-independence can be tested through a Cox model for the truncation time (Vakulenko-Lagun et al. 2022; Wang et al. 2024), but fitting a regression model for the bivariate truncation distribution under double truncation is more complex, prone to model misspecification, and cannot be done through standard methods. The second key condition is that the sampling (non-truncation) probabilities are strictly positive across the entire event time distribution, commonly known as the positivity assumption. This assumption depends on the study design (Rennert and Xie 2018, supplementary material). In situations where this assumption may be violated, e.g. in Sect. 5, there are no available methods to assess the potential impact on inference results from doubly truncated Cox regression (but see Vakulenko-Lagun et al. (2020) for right truncation).

Furthermore, the use of inverse probability weights can make these estimators highly sensitive to extreme event times. The reason is that long survivors, who appear in many risk sets of the partial likelihood and so are already potential influential points, typically have a low estimated probability of being observed. Therefore they also tend to have larger IPW weights, which increases their influence over the fitted model even further. Thus a relatively small fraction of the sample may have undue influence on the Cox model estimates, and this can lead to increased variance in small samples, as well as substantial bias if these influential points are not representative of the target population, e.g. as a result of data contamination. In practice, distinguishing between contaminated and representative samples is often challenging if not impossible, which has motivated the development of robust estimators intended to reduce the influence of such outliers on the model estimates. Several modifications of the partial likelihood score function have been proposed to improve robustness for untruncated data, e.g. Sasieni (1993a) and Sasieni (1993b), but robust estimation has not yet been explored in doubly truncated data.

Finally, all the aforementioned methods for doubly truncated data rely on bootstrap resampling for calculating standard errors. There are several drawbacks to relying on resampling methods for inference with doubly truncated survival data, however. First, the NPMLE does not have a general closed-form expression, and must be computed by iterative methods. Thus, even basic nonparametric analyses could become fairly computationally intensive in large samples due to needing to re-fit the NPMLE. In addition, the NPMLE is well-defined only under certain conditions on the data (Xiao and Hudgens 2019), so it may not be identifiable for an arbitrary subsample of the data. Finally, as already mentioned, a key assumption when using inverse probability weighted estimators is that the sampling probability is strictly positive across all event times. This positivity assumption is not verifiable using solely the observed data, however, and in Sect. 5 we find evidence that it may not hold for the AIDS data. Therefore it is good practice to perform sensitivity analysis for possible positivity violations, which involves fitting several coefficient estimators (see Sect. 2.3), and using the nonparametric bootstrap to form standard errors for each of these estimators can take an unnecessarily long time. Such issues do not typically arise in the analysis of untruncated data.

### Contributions

In this article, we study a new class of robust inverse probability weighted (IPW) Cox regression estimators that use weights based on the NPMLE, with an emphasis on deriving closed-form standard errors. This permits regression analysis without any unnecessary parametric assumptions on the truncation times, whose distribution is typically not of direct interest. As a preliminary, we derive a simplified form for the plug-in estimator of the influence function of the NPMLE and prove that it is well-defined as long as the NPMLE is identifiable. Therefore, our results also facilitate general nonparametric analysis of doubly truncated survival data, as illustrated by several examples provided in Sect. 3.1. For Cox regression we study the standard IPW partial likelihood estimator as well as a class of robust alternatives that use time-varying weights. In particular, we propose novel IPW estimators with time-varying weights based on the estimated survival function. This has the intuitive appeal of assigning relative importance to each event based on the size of its inverse probability weighted risk set, producing a highly robust estimator. For example, our simulation results in Sect. 4 show that the proposed survival function weights lead to a drastic reduction in mean squared error compared to the existing IPW estimators of Rennert and Xie (2018) and Mandel et al. (2018) under potential data contamination.

We also extend these IPW estimators to allow sensitivity analysis of the positivity assumption on the sampling probabilities and propose a simple nonparametric test and graphical diagnostic for the quasi-independent truncation assumption. We further provide closed-form standard errors that are both simple to compute through standard software and consistent under model misspecification. This is essential for sensitivity analysis of the positivity assumption, since the degree of the true positivity violation is unknown to the analyst. Theoretical properties of the estimators are developed, and we demonstrate the robustness of the proposed estimators in the presence of outliers through several simulation settings. Finally, we illustrate our proposed estimators using an AIDS study.

### Related work

Assuming quasi-independence between the truncation and event times, Efron and Petrosian (1999) and Shen (2010) provided an estimation procedure and theoretical results for the NPMLE of the event and truncation time distribution functions, with standard errors later derived by Emura et al. (2015). Also, de Uña-Álvarez and Keilegom (2021) derived the influence function for the NPMLE and suggested a plug-in estimate. This estimate is cumbersome to implement, however, and its empirical performance was not evaluated.

Both Rennert and Xie (2018) and Mandel et al. (2018) proposed Cox regression estimators that account for double truncation bias by introducing inverse probability weights in the partial likelihood score function. They relied on the bootstrap for variance estimation when using NPMLE weights, however, since they were not able to derive closed-form standard errors. Later, Rennert and Xie (2022) proposed a conditional maximum likelihood estimator (MLE) that relaxed the quasi-independent truncation assumption to covariate-dependent truncation for the Cox model, also relying on bootstrap for inference (note their conditional MLE approach was initially deposited in a preprint server in 2018: https://arxiv.org/abs/1803.09830). Conditional maximum likelihood estimation has further been extended to doubly truncated data to fit semiparametric transformation models (Shen and Hsu 2020) and to analyze data that is also interval-censored (Shen 2025). If the stronger assumption of quasi-independent truncation is valid, however, IPW estimators with NPMLE weights hold several practical advantages over the MLE. First, they can be computed directly through standard Cox regression software once the weights are obtained, as described in Sect. 2.4, while the MLE uses an iterative EM algorithm. In fact, this ease of implementation also applies to our proposed standard errors (see Remark 3). Finally, IPW estimators can directly use standard Cox model diagnostics for model misspecification and non-proportional hazards based on plots of weighted residuals, e.g. Grambsch and Therneau (1994), which are widely available in standard software.

### Organization

We describe the proposed doubly truncated Cox regression methods in Sect. 2, with the nonparametric test and graphical diagnostic for the quasi-independence assumption in Sect. 2.1, the proposed class of IPW estimators with time-varying weights in Sect. 2.2, and the sensitivity analysis for positivity violations in Sect. 2.3. The theoretical results, including closed-form standard errors for all proposed methods, are provided in Sect. 3. In Sect. 3.1 we describe the developments for the NPMLE, while Sect. 3.2 contains the asymptotics for the IPW estimators. The proposed methods are evaluated through extensive simulations in Sect. 4, and illustrated through an application to an AIDS dataset in Sect. 5.

## Proposed methods

For any *p*-vector ${\varvec{a}}$, let ${\varvec{a}}^{\otimes 0} = 1$, ${\varvec{a}}^{\otimes 1} = {\varvec{a}}$, and ${\varvec{a}}^{\otimes 2} = {\varvec{a}}{\varvec{a}}^{\textsf{T}}$. Furthermore, operations such as $1/{\varvec{a}}$ and ${\varvec{a}}^2$ are understood to be applied elementwise, and ${diag}({\varvec{a}})$ denotes the $p\times p$ diagonal matrix with *i*th diagonal entry equal to ${\varvec{a}}_i$. For any matrix ${\varvec{A}}$, ${\varvec{A}}_{ij}$ denotes its (*i*, *j*)th entry. ${\varvec{I}}_n$ denotes the $n\times n$ identity matrix, while ${\varvec{1}}_n$ and ${\varvec{0}}_n$ are *n*-vectors of ones and zeros, respectively. If *X* is a random variable, then its support is denoted by $[a_X, b_X]$.

For a random event time *T*, we define the at-risk process $Y(t) = {\varvec{1}}(T\ge t)$, the counting process $N(t) = {\varvec{1}}(T\le t)$, and the potentially time-varying *p*-dimensional covariate vector ${\varvec{x}} (t)$ for $t\in [a_T,b_T]$. We also assume that, prior to truncation, the data follows a Cox regression model with hazard function

$$\begin{aligned} \lambda (t|\{{\varvec{x}} (s),s\le t\}) = \exp \left( {\varvec{x}} (t)^{\textsf{T}}\varvec{\beta }^0\right) \lambda _0(t), \end{aligned}$$

where $\varvec{\beta }^0$ is the true regression parameter and $\Lambda _0(t) = \int _{-\infty }^t \lambda _0(s)ds$ is the cumulative baseline hazard function. We use ${\mathbb {P}}$ to denote the law of the pre-truncation data. Since the left and right truncation times (*U*, *V*) are assumed to be jointly independent of the event time (and covariates) within the observable data region $U\le T\le V$, the non-truncation probability at event time *t* is $\pi (t) = {\mathbb {P}}(U\le T\le V|T=t, {\varvec{x}} (\cdot )) = {\mathbb {P}}(U\le t\le V)$.

We observe a random sample of doubly truncated data $\{T_i, U_i, V_i, {\varvec{x}}_{i}(\cdot )\}$, $i=1,\ldots ,n$, conditional on $T_i\in [U_i, V_i]$. For example, in the AIDS data analyzed in Sect. 5, $T_i$ is the *i*th AIDS incubation time, $U_i$ is the *i*th AIDS discovery time (1982), and $V_i$ is the *i*th end of study time (1986). Note that although the truncation times are fixed calendar dates in this case, $(U_i,V_i)$ do vary across individuals since time is measured from the date of HIV infection. Let ${\mathbb {P}}^*$ be the law of the doubly truncated observed data, as opposed to the pre-truncation law ${\mathbb {P}}$. All probabilistic statements are understood to be with respect to this post-truncation distribution unless specified otherwise. By the quasi-independence between (*U*, *V*) and *T*, the pre-truncation event time cumulative distribution function (CDF) can be expressed as $F(t) = \int _{a_T}^t a(s)^{-1}dF^*(s)$, where $F^*(t) = {\mathbb {P}}^*(T\le t)$ is the post-truncation CDF, $a(t) = \pi (t)/\alpha$, and $\alpha = {\mathbb {P}}(U\le T\le V) = [\int \pi (t)^{-1} dF^*(t)]^{-1}$ is the overall non-truncation probability.

### Nonparametric diagnostic for the quasi-independent truncation assumption

Consistency of the IPW estimators considered in this paper depends critically on the quasi-independent truncation assumption, which states that $\{T,{\varvec{x}} (\cdot )\}$ is independent of (*U*, *V*) within the observable region $U\le T\le V$ under ${\mathbb {P}}$. If this assumption holds, the selection probabilities for $\{T,{\varvec{x}} (\cdot )\}$ reduce to ${\mathbb {P}}(U\le T\le V|T=t)$ and can be estimated consistently by the NPMLE. This assumption may be violated if, for example, the truncation times and event time are associated with common baseline risk factors. Therefore we propose a two-step nonparametric diagnostic for assessing the quasi-independence assumption.

First, divide the data into *S* covariate strata. These strata should be based on binned values of covariates that are hypothesized to potentially be associated with the truncation times, and the diagnostic may be repeated using different stratifications. Since each additional stratum tends to reduce the power of the test, we recommend using only 2-4 strata in practice based on our simulation studies (Online Resource Section S5.2). Then, test for quasi-independent truncation within each stratum using, for example, conditional Kendall’s tau (Martin and Betensky 2005). This checks that the *s*th stratum-specific selection probabilities ${\mathbb {P}}(U\le T\le V|T=t; s)$ reduce to $\pi (t|s)={\mathbb {P}}(U\le t\le V|s)$.

Finally, estimate the stratum-specific selection probabilities $\pi (t|s)$, $s=1,\ldots ,S$, by computing the NPMLE within each stratum. Plot these estimates ${\widehat{\pi }}(t|s)$ against time, along with the unstratified NPMLE estimates ${\widehat{\pi }}(t)$. Under quasi-independent truncation, the truncation time distribution should not vary by stratum, so we should have $\pi (t|s) = \pi (t)$ for all $t\in [a_T,b_T]$ and $s=1,\ldots ,S$. Visually, this can be assessed by how close the stratum-specific estimates ${\widehat{\pi }}(t|s)$ are to the unstratified estimates ${\widehat{\pi }}(t)$, and the magnitude of these deviations quantify the estimated departure from quasi-independent truncation. In Online Resource Section S2, we derive the asymptotic null distribution of the test statistic $\sup _{t,s} |{\widehat{\pi }}(t|s) - {\widehat{\pi }}(t)|$ and describe how to compute a p-value and confidence band for the diagnostic plot based on sampling from this distribution, using closed-form estimates. Furthermore, we assess the power and Type I error rate of this diagnostic test through several simulation settings in Online Resource Section S5.2. See Sect. 5.1 for an illustration of this diagnostic when applied to the AIDS data.

### Robust IPW Cox regression

In this section, we briefly review existing IPW estimators for doubly truncated Cox regression, and then introduce our proposed estimators that are robust to outliers in the event time.

Since the truncated data is generally a biased sample, the classical maximum partial likelihood estimator is no longer a consistent estimator for the regression parameters $\varvec{\beta }^0$. One can correct for this bias, however, by weighting each individual in the sample proportional to the inverse probability of not being truncated, e.g. using weights $a(T_i)^{-1}=\pi (T_i)^{-1}\alpha$. Intuitively, individuals with a low probability of being observed are given larger weights and the resulting pseudo-population $\{a(T_i)^{-1}N_i(t), a(T_i)^{-1}Y_i(t)\}$ is an approximately representative sample of the pre-truncation distribution. In order to avoid unnecessary parametric assumptions or modeling of the truncation distribution, we consider inverse probability weighting using the NPMLE of the weights, which can be obtained from the point masses of the NPMLE of the event time CDF (see Sect. 2.4). Let $\widehat{a}(T_1)^{-1},\ldots ,\widehat{a}(T_n)^{-1}$ denote the estimated weights. de Uña-Álvarez and Keilegom (2021) showed that $\widehat{a}(t)$ converges in probability to *a*(*t*) uniformly in *t* under some assumptions.

Assuming no tied event times, the standard inverse probability weighted (IPW) partial likelihood has the score function

$$\begin{aligned} \nabla \varvec{\ell }(\varvec{\beta };\varvec{\widehat{a}})= \sum _{i=1}^n \int _{a_T}^{b_T}\widehat{a}(T_i)^{-1}\left[ {\varvec{x}}_{i}(t) - \varvec{\overline{x}}_{\varvec{\widehat{a}}}(t,\varvec{\beta })\right] dN_i(t), \end{aligned}$$

where

$$\begin{aligned} \varvec{\overline{x}}_{\varvec{\widehat{a}}}(t,\varvec{\beta }) = \frac{{\varvec{S}}_{\varvec{\widehat{a}}}^{(1)}(t,\varvec{\beta })}{{\varvec{S}}_{\varvec{\widehat{a}}}^{(0)}(t,\varvec{\beta })}, \ {\varvec{S}}_{\varvec{\widehat{a}}}^{(r)}(t,\varvec{\beta }) = \frac{1}{n}\sum _{j=1}^n \widehat{a}(T_j)^{-1}Y_j(t){\varvec{x}}_{j}(t)^{\otimes r}\exp \left( {\varvec{x}}_{j}(t)^{\textsf{T}}\varvec{\beta }\right) ,\ \end{aligned}$$

for $r=0,1,2$. This inverse probability weighted partial likelihood has been previously applied to survey data, where the weights are known (Binder 1992; Lin 2000), for general biased samples with weights estimated parametrically (Pan and Schaubel 2008), and specifically to account for double truncation (Rennert and Xie 2018).

An alternative approach proposed by Mandel et al. (2018), which we refer to as stabilized weighting, only applies weights to individuals in the risk set covariate averages $\varvec{\overline{x}}_{\varvec{\widehat{a}}}(t,\varvec{\beta })$ of the score function, but still results in an unbiased estimating function. We consider this method, as well as other potentially robust alternatives to the IPW partial likelihood, under the general framework of time-varying weights with the structure $\widehat{w}_i(t) = \widehat{w}(t)/\widehat{a}(T_i)$, e.g. with $\widehat{w}(t) = \widehat{a}(t)$ for stabilized weights. The use of such weights can be considered an inverse probability weighted analogue of what is known in the untruncated data setting as weighted Cox regression (Schemper 1992; Sasieni 1993a; Schemper et al. 2009). It can be particularly useful for doubly truncated data because the standard maximum partial likelihood estimator is known to be sensitive to extreme event times, and the introduction of inverse probability weights naturally tends to increase the influence of such event times, which generally have a low probability of being observed, even further. The IPW partial likelihood with time-varying weights based on weight function $\widehat{w}(\cdot )$ has the score function

$$\begin{aligned} \nabla \varvec{\ell }(\varvec{\beta };\varvec{\widehat{a}}, \widehat{w})= \sum _{i=1}^n \int _{a_T}^{b_T} \frac{\widehat{w}(t)}{\widehat{a}(T_i)}\left[ {\varvec{x}}_{i}(t) - \varvec{\overline{x}}_{\varvec{\widehat{a}}}(t,\varvec{\beta })\right] dN_i(t), \end{aligned}$$

with its derivative denoted as $\nabla ^2 \varvec{\ell }(\varvec{\beta };\varvec{\widehat{a}}, \widehat{w})$. Now let ${\varvec{s}}_{{\varvec{a}}}^{(r)}(t,\varvec{\beta }) = {\mathbb {E}}^*[{\varvec{S}}_{{\varvec{a}}}^{(r)}(t,\varvec{\beta })]$ and ${\varvec{e}}_{{\varvec{a}}}(t,\varvec{\beta }) = {\varvec{s}}_{{\varvec{a}}}^{(1)}(t,\varvec{\beta })/{\varvec{s}}_{{\varvec{a}}}^{(0)}(t,\varvec{\beta })$. One can show algebraically that ${\mathbb {E}}^*[{\varvec{x}} (t)|T=t] = {\varvec{e}}_{{\varvec{a}}}(t,\varvec{\beta }^0)$, the uniform limit of $\varvec{\overline{x}}_{\varvec{\widehat{a}}}(t,\varvec{\beta }^0)$, so $n^{-1}\nabla \varvec{\ell }(\varvec{\beta }^0;\varvec{\widehat{a}}, \widehat{w})\rightarrow _p {\varvec{0}}_p$ under the theoretical assumptions outlined in Sect. 3. Thus the score function $\nabla \varvec{\ell }(\varvec{\beta };\varvec{\widehat{a}}, \widehat{w})$ provides an approximately unbiased estimating equation for $\varvec{\beta }^0$ under a wide class of non-negative weight functions $\widehat{w}(\cdot )$, intuitively because the weighting in the risk-set average $\varvec{\overline{x}}_{\varvec{\widehat{a}}}(t,\varvec{\beta })$ is unaffected. Standard errors for this class of IPW estimators can be computed with standard software and are provided in Sect. 3.2.

Some options for the weight function $\widehat{w}(\cdot )$ include:

1. Stabilized weights: $$\widehat{w}(t) = \widehat{a}(t)$$
2. Survival function: $$\widehat{w}(t) = 1-\widehat{F}(t)$$
3. Fleming-Harrington weights: $\widehat{w}(t) = \widehat{F}(t)^r[1-\widehat{F}(t)]^{s}$ for $r,s\ge 0$
4. Combinations of above, e.g. stabilized survival $\widehat{w}(t) = \widehat{a}(t)[1-\widehat{F}(t)]$.

In order to produce an estimator that is robust to outliers in the event time, the weight function should de-emphasize unusually large event times, where the risk set averages in the score function are dominated by a small number of individuals. We advocate for survival function weighting, which accomplishes this goal in the following intuitive manner: with $\widehat{w}(t) = 1-\widehat{F}(t)$, each event is weighted by the number of individuals at-risk in the IPW pseudo-sample at the given event time, since $1-\widehat{F}(t) \propto \sum _i {\varvec{1}}(T_i>t )\widehat{a}(T_i)^{-1}$. If the estimated sampling probabilities are observed to approach zero, this can be combined with stabilized weighting to further reduce the variance of the coefficient estimates. Stabilized weights alone, however, may not sufficiently protect against influential points. Intuitively this is because the sampling probability is a functional of only the truncation time distribution, so the shape of *a*(*t*) does not change under different event time distributions. This is also supported by our simulation results in Sect. 4, where the IPW estimator using the stabilized weights of Mandel et al. (2018) is shown to be much more sensitive to data contamination compared to using the proposed survival function weights.

Lastly, given coefficient estimates $\varvec{\widehat{\beta }}$, the inverse probability weighted estimator for the cumulative baseline hazard is

$$\begin{aligned} {\widehat{\Lambda }}_{0}(t;\varvec{\widehat{\beta }},\varvec{\widehat{a}}) = \frac{1}{n}\sum _{i=1}^n \int _{-\infty }^t \frac{\widehat{a}(T_i)^{-1}dN_i(s)}{{\varvec{S}}_{\varvec{\widehat{a}}}^{(0)}(s,\varvec{\widehat{\beta }})}. \end{aligned}$$

Note that the form of the estimator is unchanged when using time-varying weights, apart from the different coefficient estimate $\varvec{\widehat{\beta }}$.

### Sensitivity analysis for potential violations of the positivity assumption on the sampling probabilities

A key condition underlying the inverse probability weighted estimators described above is that all event times have a positive probability of being observed, i.e. $\inf _{t\in [a_T,b_T]}a(t)>0$. Since this condition cannot be assessed using solely the observed data, it is generally good practice to conduct a sensitivity analysis in order to quantify the potential impact of positivity violations on the coefficient estimates. We extend the approach of Vakulenko-Lagun et al. (2020), which was developed for right-truncated data, to double truncation. Due to the complexity of the conditional survival function under time-varying covariates, we only consider time-independent covariates here.

Suppose that the observed event time distribution is restricted to the deterministic interval [*L*, *R*] due to zero sampling probability outside this interval, with $L>a_T$ and $R<b_T$ which results in a violation of the positivity assumption. This positivity violation introduces another layer of bias in the sample, in addition to the bias from the random truncation intervals. Without further assumptions, inference must be done conditional on $T\ge L$ and $T\le R$. The first condition alone is less problematic for Cox regression because the hazard rate conditional on $T\ge L$ is simply ${\varvec{1}}(t\ge L)\lambda (t|{\varvec{x}})$, which is unchanged at any time in the observable region $t\ge L$, so hazard ratio estimation is unaffected. Intuitively this is because the hazard rate conditions on survival up to time *t*. The second condition $T\le R$, however, can change the hazard rate at all $t\le R$, so it will generally result in biased estimates.

In order to correct the bias from the positivity violation, we first quantify the degree of the positivity violation by the truncated baseline probability mass $p_{r|l} = {\mathbb {P}}(T> R|T> L,{\varvec{x}}={\varvec{0}}_p) = S_0(R)/S_0(L)$, where $S_0(t)$ is the true baseline survival function. If $p_{r|l}$ were known, we could impute the follow-up time that was truncated due to non-positivity by defining the modified at-risk process $\widetilde{Y}_i(t;\varvec{\beta },p_{r|l}) = Y_i(t) + p_{r|l}^{\exp ({\varvec{x}}_i^{\textsf{T}}\varvec{\beta })}/ (1-p_{r|l}^{\exp ({\varvec{x}}_i^{\textsf{T}}\varvec{\beta })} )$. It is straightforward to show that ${\mathbb {E}}[ \widetilde{Y}(t;\varvec{\beta }^0,p_{r|l})| T\in [L,R],{\varvec{x}}] \propto S_0(t)^{\exp ({\varvec{x}}^{\textsf{T}}\varvec{\beta }^0)} = {\mathbb {E}}[Y(t)|{\varvec{x}}]$ for any $t\in [L,R]$, which accounts for the bias due to non-positivity within the risk-set average. Letting $\varvec{\widetilde{x}}_{\varvec{\widehat{a}}}(t,\varvec{\beta })$ be the risk-set average based on $\widetilde{Y}_i(t;\varvec{\beta },p_{r|l})$, the regression coefficients would be estimated using the modified IPW score function

$$\begin{aligned} {\varvec{r}}(\varvec{\beta };\varvec{\widehat{a}}, \widehat{w})= \sum _{i=1}^n \int _{a_T}^{b_T} \frac{\widehat{w}(t)}{\widehat{a}(T_i)}\left[ {\varvec{x}}_i - \varvec{\widetilde{x}}_{\varvec{\widehat{a}}}(t,\varvec{\beta })\right] dN_i(t). \end{aligned}$$

The true truncated probability mass is not known in practice, since it involves the true baseline hazard, and estimating it jointly with $\varvec{\beta }^0$ may not be feasible. One can instead obtain a range of plausible coefficient estimates by fitting estimates $\varvec{\widehat{\beta }}_{w,p_{r|l}}$ along a fixed grid of values for $p_{r|l}$. A sensitivity interval (SI) for $\varvec{\beta }^0$ assuming truncated mass of at most *q* is then given by the union of the confidence intervals from $\{\varvec{\widehat{\beta }}_{w,p_{r|l}}:\ p_{r|l}\le q\}$. The standard errors needed to construct these confidence intervals are provided in Sect. 3.2.

### Software implementation

To estimate the pre-truncation event time CDF, consider the class of right-continuous step functions with positive increments $\phi _i$ at the observed event times $T_i$. The NPMLE is $\widehat{F}(t) = \sum _{i=1}^n {\varvec{1}}(T_i\le t){\widehat{\phi }}_i$, where the point masses $\varvec{{\widehat{\phi }}} = ({\widehat{\phi }}_1\ldots ,{\widehat{\phi }}_n)^{\textsf{T}}$ maximize the nonparametric likelihood

$$\begin{aligned} \prod _{i=1}^n\frac{\phi _i}{\Phi _i},\ \varvec{\Phi } = (\Phi _1,\ldots ,\Phi _n)^{\textsf{T}} = {\varvec{J}}^{\textsf{T}}\varvec{\phi }, \ {\varvec{J}}_{ij} = {\varvec{1}}(T_i\in [U_j,V_j]) \end{aligned}$$

subject to the constraints $\phi _i>0$, $i=1,\ldots ,n$, and $\sum _{i=1}^n \phi _i = 1$ (Efron and Petrosian 1999). It can be shown that the point masses of $\widehat{F}$ are inversely proportional to the estimated selection probabilities, that is ${\widehat{\phi }}_i = (n\widehat{a}(T_i))^{-1}$ where $\{\widehat{a}(T_i)^{-1},\ i=1,\ldots ,n\}$ are the estimated inverse probability weights which are used in the IPW estimators discussed above.

The NPMLE is commonly computed by an EM algorithm that iteratively updates the estimates $\varvec{\phi }^{(k)}$ by setting $\varvec{\Phi }^{(k)} = {\varvec{J}}^{\textsf{T}}\varvec{\phi }^{(k)}$ and $1/\varvec{\phi }^{(k+1)} = {\varvec{J}}(1/\varvec{\Phi }^{(k)})$, then normalizing $\varvec{\phi }^{(k+1)}$ to sum to one (Efron and Petrosian 1999).

The proposed IPW estimators can be fit using standard software packages for Cox regression. In the R programming language (R Core Team 2023, v4.3.1), for example, one can use the coxph function from the survival package (Therneau and Grambsch 2000; Therneau 2023, v3.5-5) with weights set to $\widehat{w}(T_i)\widehat{a}(T_i)^{-1}$ and an offset of $-\log (\widehat{w}(T_i))$ to obtain an IPW estimator with time-varying weights. The offset term produces the correct risk set averages without $\widehat{w}(T_i)$, since $n^{-1}\sum _i \widehat{w}(T_i)\widehat{a}(T_i)^{-1}Y_i(t){\varvec{x}}_{i}(t)^{\otimes r}\exp ({\varvec{x}}_{i}(t)^{\textsf{T}}\varvec{\beta }-\log \widehat{w}(T_i)) = {\varvec{S}}_{\varvec{\widehat{a}}}^{(r)}(t,\varvec{\beta })$. The model components needed to compute the standard errors are also simple to extract this way (see Remark 3). This convenient workaround will, however, produce an incorrect baseline hazard estimate due to the presence of $\widehat{w}(t)$ in the estimated hazard increments. To create a model object that will produce the correct hazard, one can then call coxph again with init set to the estimated coefficients, weights of $\widehat{a}(T_i)^{-1}$ (free of $\widehat{w}(T_i)$), and control set to coxph.control(iter.max = 0) to not update the coefficient estimates further. Lastly, all standard errors should be adjusted to account for the variability of the NPMLE as described in Sect. 3 below.

## Theoretical results

We make the following assumptions:

1. Positivity: $\inf _{dK^*(u,v)>0}{\mathbb {P}}(T\in [u.v])>\delta _1$, where $K^*(u,v)={\mathbb {P}}^*(U\le u,V\le v)$, and $\inf _{t\in [a_T,b_T]} \pi (t)>\delta _2$ hold for some constants $\delta _1>0$ and $\delta _2>0$. In addition:
  - We have $a_T<a_{V_1}$, $b_{U_1}<b_T$, $b_T-a_T<\infty$, and ${\mathbb {P}}^*(V-U\ge \gamma )=1$ for some $\gamma >0$.
  - The marginal post-truncation densities of *U* and *V* are bounded on $[a_T,b_T]$, where *U* and *V* are assumed to be absolutely continuous.
2. NPMLE identifiability: the directed graph with edges defined by $i\rightarrow j$ if $T_j\in [U_i, V_i]$ is strongly connected (for any *i* and *j*, there exists a path from *i* to *j* and from *j* to *i*). This condition is due to Xiao and Hudgens (2019) and can be easily checked with standard software.
3. Quasi-independent truncation: $\{T,{\varvec{x}} (\cdot )\}$ is independent of (*U*, *V*) within the observable region $U\le T\le V$, under ${\mathbb {P}}$.
4. Bounded covariates: $\max _{j=1,\ldots ,p} (|{\varvec{x}} (a_T)_j| + \int _{a_T}^{b_T} |d{\varvec{x}} (t)_j|)$ is bounded above almost surely.
5. Monotone hazard: $\Lambda _0(t) = \int _{-\infty }^t \lambda _0(t)dt$ where $\lambda _0(t)$ is continuous and strictly positive on $[a_T,b_T]$.
6. The matrix $\varvec{\mathcal {I}}_w = {\mathbb {E}} [\int _{a_T}^{b_T} w(t) \{{\varvec{s}}_{{\varvec{a}}}^{(2)}(t,\varvec{\beta }^0){\varvec{s}}_{{\varvec{a}}}^{(0)}(t,\varvec{\beta }^0)^{-1} - {\varvec{e}}_{{\varvec{a}}}(t,\varvec{\beta }^0)^{\otimes 2} \} dN(t) ]$ is positive definite.
7. For time-varying weights, the weight function $\widehat{w}(t)$ converges in probability to a deterministic function $w(t)\ge 0$ of bounded variation and $\widehat{w}(t) - w(t) = n^{-1}\sum _{i=1}^n L_i^w(t) + o_p\left( 1/\sqrt{n}\right)$ uniformly in $t\in [a_T,b_T]$, for some function $L_i^w(t)$ depending on only the *i*th data point.

The positivity assumption (Assumption 1) and the quasi-independent truncation assumption (Assumption 3) allow nonparametric estimation of the inverse probability weights (de Uña-Álvarez and Keilegom 2021). We propose a diagnostic test for the quasi-independent truncation assumption in Sect. 2.1. The positivity assumption depends on the study design (recruitment period, study duration, inclusion criteria) and may be checked using external information on the range of possible event times (see Sect. 5). In cases where such information is not available or when it suggests that the positivity assumption is invalid, the potential impact of a positivity violation can be assessed by our sensitivity analysis proposed in Sect. 2.3. The graphical condition in Assumption 2 can be easily checked with standard software. The remaining Assumptions 4-7 are mild and commonly applied in Cox regression. In particular, Assumption 7 holds for both the existing and proposed estimators described in Sect. 2.2.

### Influence function estimation for the NPMLE

Recently, de Uña-Álvarez and Keilegom (2021) derived the influence function for the NPMLE of the event time distribution function $\widehat{F}(t)$, which involves an infinite sum of bounded linear operators. Since an estimate of this influence function can be used to compute standard errors for $\widehat{F}(t)$ and $\widehat{a}(t)$, they suggested estimating the infinite sum by plugging in the corresponding sample quantities and truncating the sum at some cutoff *c*. This can be fairly computationally intensive, however, and we further show in Lemma 1 below that the exact plug-in estimate has a simple closed-form which exists whenever the NPMLE is identifiable in the observed sample (Assumption 2).

Under Assumptions 1 and 3, the results of de Uña-Álvarez and Keilegom (2021) imply that

$$\begin{aligned} \widehat{F}(t) - F(t) = \frac{1}{n}\sum _{i=1}^n \mathcal {A}[h_i](t) + o_p\left( 1/\sqrt{n}\right) \end{aligned}$$

where $\mathcal {A}$ is a bounded linear operator with $\mathcal {A}[h](t) = \sum _{r=0}^\infty A^r[h](t)$ for

$$\begin{aligned} A[h](t) = \int _{a_T}^t \int _{u\le s\le v} \frac{h(v) - h(u-)}{(F(v) - F(u-))^2} dK^*(u,v) \frac{dF^*(s)}{a(s)^2} \end{aligned}$$

and $h_i(t)$ is a function that depends on the *i*th data point

$$\begin{aligned} h_i(t) = \frac{{\varvec{1}}(T_i\le t)}{a(T_i)} - \frac{{\varvec{1}}(U_i\le t)}{F(V_i) - F(U_i-)}\int _{a_T}^t {\varvec{1}}(s\in [U_i,V_i])\frac{dF^*(s)}{a(s)^2}. \end{aligned}$$

For $\widehat{a}(t)$ they derived

$$\begin{aligned} \widehat{a}(t) - a(t) = \frac{1}{n}\sum _{i=1}^n \left[ \frac{{\varvec{1}}(t\in [U_i,V_i])}{F(V_i) - F(U_i-)} - a(t) - \widetilde{\mathcal {A}}[h_i](t) \right] + o_p\left( 1/\sqrt{n}\right) \end{aligned}$$

with

$$\begin{aligned} \widetilde{\mathcal {A}}[h](t) = \int _{u\le t\le v} \frac{\mathcal {A}[h](v) - \mathcal {A}[h](u-)}{[F(v) - F(u-)]^2}dK^*(u,v).\end{aligned}$$

Here $K^*(u,v) = {\mathbb {P}}^*(U\le u, V\le v)$, and the plug-in estimates for $F^*$ and $K^*$ would be the corresponding empirical distribution functions from the observed sample. Then the estimates for $A[h_i](t)$ and $h_i(t)$ are right continuous step functions with jumps at the observed event times.

In order to estimate $\mathcal {A}[h_i](t)$, first note that the plug-in estimator for $h_j(T_i)$ is given by

$$\begin{aligned} {\varvec{H}}_{ij} = n{\widehat{\phi }}_j{\varvec{1}}(T_j\le T_i) - \frac{n\sum _{k=1}^n {\varvec{J}}_{kj}{\varvec{1}}(T_k\le T_i){\widehat{\phi }}_k^2}{{\widehat{\Phi }}_j}. \end{aligned}$$

Also, denote the $n\times n$ lower triangular matrix of ones by ${\varvec{S}}$, so ${\varvec{S}}_{ij} = {\varvec{1}}(i\ge j)$. Lastly, define the matrices

$$\begin{aligned} \varvec{\widetilde{A}} = {\varvec{J}}{diag}(1/\varvec{{\widehat{\Phi }}}^2){\varvec{J}}^{\textsf{T}}{\varvec{S}}^{-1}/n \end{aligned}$$

and

$$\begin{aligned} {\varvec{A}} = n{\varvec{S}}{diag}(\varvec{{\widehat{\phi }}}^2)\varvec{\widetilde{A}} = {\varvec{S}}{diag}(\varvec{{\widehat{\phi }}}^2){\varvec{J}}{diag}(1/\varvec{{\widehat{\Phi }}}^2){\varvec{J}}^{\textsf{T}}{\varvec{S}}^{-1}. \end{aligned}$$

With sorted event times, $({\varvec{A}}{\varvec{H}})_{ji}$ is the plug-in estimate of $A[h_i](T_j)$ when there are no ties. If there are ties, we define the $n\times n$ matrix ${\varvec{I}}^u$ with *j*th row equal to the *k*th standard basis vector ${\varvec{e}}_k$, where $T_k=T_{j}$ and either $k=n$ or $T_{k+1}\ne T_j$. Then it is straightforward to verify that $({\varvec{I}}^u{\varvec{A}}{\varvec{H}})_{ji}$ is the plug-in estimate of $A[h_i](T_j)$. Lemma 1 below provides estimates for the influence functions of $\widehat{F}(t)$ and $1/\widehat{a}(t)$, evaluated at $T_1,\ldots ,T_n$.

#### Lemma 1

(Estimator for the influence function of the NPMLE) Suppose the event times are sorted so that $T_1\le T_2\le \ldots \le T_n$ and assume the strong connectedness condition holds. Let ${\varvec{B}} = {\varvec{S}}{diag}(\varvec{{\widehat{\phi }}})$, ${\varvec{C}} = [{\varvec{I}}_{n-1}\ -\!{\varvec{1}}_{n-1}]^{\textsf{T}}$, and $\varvec{\mathcal {A}}$ be the matrix of plug-in influence function estimates for $\widehat{F}$, such that $\varvec{\mathcal {A}}_{ji}$ estimates $\mathcal {A}[h_i](T_{j})$. Then

$$\begin{aligned} \varvec{\mathcal {A}} = {\varvec{I}}^u{\varvec{B}}{\varvec{C}}\left[ {\varvec{C}}^{\textsf{T}}{\varvec{B}}^{-1}\left( {\varvec{I}}_n - {\varvec{A}}\right) {\varvec{B}}{\varvec{C}} \right] ^{-1}{\varvec{C}}^{\textsf{T}}{\varvec{B}}^{-1}{\varvec{H}} \end{aligned}$$

and the plug-in influence function estimates for $\widehat{a}(t)$, evaluated at $t=T_j$ and the *i*th data point, are

$$\begin{aligned} \frac{{\varvec{J}}_{ji}}{{\widehat{\Phi }}_i} - \widehat{a}(T_j) - (\varvec{\widetilde{A}}\varvec{\mathcal {A}})_{ji}. \end{aligned}$$

Furthermore, letting ${\varvec{L}}$ be the matrix of influence function estimates for $1/\widehat{a}(t)$,

$$\begin{aligned} {\varvec{L}}_{ji} = -\frac{1}{\widehat{a}(T_j)^2}\left[ \frac{{\varvec{J}}_{ji}}{{\widehat{\Phi }}_i} - \widehat{a}(T_j) - (\varvec{\widetilde{A}}\varvec{\mathcal {A}})_{ji}\right] . \end{aligned}$$

is the estimate corresponding to $t=T_j$ and the *i*th data point.

The proof for Lemma 1 is provided in Online Resource Section S1.

Since the estimated influence function for $\widehat{F}(t)$ is a right-continuous step function with jumps at the observed event times, $\varvec{\mathcal {A}}$ can be used for covariance estimation of $\widehat{F}(t)$ at any time points. The estimate ${\varvec{L}}$, on the other hand, would have right-continuous jumps at observed left truncation times, and left-continuous jumps at right truncation times. Therefore it should be evaluated at additional time points, which is straightforward, when conducting nonparametric analysis of the weight process. Only ${\varvec{L}}$ is required to construct standard errors for the IPW estimators, as described in Sect. 3.2 below.

The utility of these simple closed-form influence function estimates extends beyond Cox regression, which is the focus of Sect. 3.2, to general nonparametric analysis of doubly truncated survival data. They allow one to obtain confidence intervals and conduct hypothesis tests based on smooth functionals of the NPMLE without needing further complicated theoretical derivations and without relying on the nonparametric bootstrap. We discuss this below in the context of testing for ignorable sampling bias, with more examples provided in Online Resource Section S3. Additional simulation results for the NPMLE are available in Online Resource Section S5.1.

#### Remark 1

(Ignorable sampling bias) The NPMLE typically has higher variance than the much simpler empirical distribution function, which does not correct for truncation bias, and the same can be said for IPW vs unweighted Cox regression estimates. Given a sample of doubly truncated data, one may wish to evaluate whether the sampling bias is ignorable, since then standard methods for untruncated data could be applied. This situation occurs when the selection probability is constant, i.e. $H_0:a(t)= 1$, $\forall t\in [a_T,b_T]$, or equivalently $F(t) = F^*(t)$. Recently﻿, de Uña-Álvarez (2023) proposed testing for ignorable sampling bias through the statistic $\sqrt{n}\sup _{t\in [a_T,b_T]}|\widehat{F}(t) - \widehat{F}^*(t)|$, where $\widehat{F}^*(t)$ is the empirical distribution function. They noted that the asymptotic null distribution of this statistic is that of the supremum of a mean-zero Gaussian process *G*(*t*) with $Cov(G(s), G(t)) = {\mathbb {E}}^*[f_i(t)f_i(s)]$ and $f_i(t) = \mathcal {A}[h_i](t) - {\varvec{1}}(T_i\le t) + F(t)$, but they declined to work with this distribution due to its complexity. Instead they relied on bootstrap resampling to approximate the null distribution. It is computationally simpler, however, to use the influence function estimates in Lemma 1 to estimate this covariance function, and then calculate a p-value by repeated simulation of the process *G*(*t*).

#### Remark 2

(Ignorable sampling bias continued) We may instead assess the null hypothesis of ignorable sampling bias directly through $\widehat{a}(t)$, using the statistic $\sqrt{n}\sup _{t\in [a_T,b_T]}|\widehat{a}(t) - 1|$. Under the null, this converges weakly to the restricted supremum of a mean-zero Gaussian process *G*(*t*) with $Cov(G(t), G(s)) = {\mathbb {E}}^*[L_i^a(t)L_i^a(s)]$, where $L_i^a(t)$ is the influence function for *a*(*t*). Using the estimates from Lemma 1, one can calculate the p-value for this test by simulation.

### Robust IPW Cox regression with time-varying weights

Let $\varvec{\widehat{\beta }}_w$ be the IPW coefficient estimates obtained using weight function $\widehat{w}(\cdot )$. Define

$$\begin{aligned} M_i(t) = N_i(t) - \int _{-\infty }^t Y_i(s)\exp ({\varvec{x}}_{i}(s)^{\textsf{T}}\varvec{\beta }^0)d\Lambda _0(s) \end{aligned}$$

for $t\in [a_T,b_T]$ and let $L_i(t)$ be the influence function of $\widehat{a}(t)^{-1}$. The plug-in estimators are $\widehat{M}_i(t) = N_i(t) - \int _{-\infty }^t Y_i(s)\exp ({\varvec{x}}_{i}(s)^{\textsf{T}}\varvec{\widehat{\beta }}_w)d{\widehat{\Lambda }}_0(s;\varvec{\widehat{\beta }}_w,\varvec{\widehat{a}})$, and $\widehat{L}_{i}(T_j) = {\varvec{L}}_{ji}$ (see Lemma 1 above).

Consistency of $\varvec{\widehat{\beta }}_w$ can be shown by a concavity argument as in Rennert and Xie (2018). Theorem 1 below provides the standard errors for $\varvec{\widehat{\beta }}_w$, while Theorem 2 provides the influence function estimates for the baseline hazard estimator.

#### Theorem 1

(Standard errors for robust IPW Cox regression with time-varying weights) Suppose $\varvec{\widehat{\beta }}_w\rightarrow _p \varvec{\beta }^0$. Under Assumptions 1-7,

$$\begin{aligned} \varvec{\widehat{\beta }}_w - \varvec{\beta }^0 = \frac{1}{n}\sum _{i=1}^n\varvec{\mathcal {I}}_w^{-1}\left( {\varvec{U}}_{w1i} + {\varvec{U}}_{w2i} + {\varvec{U}}_{w3i} \right) + o_p\left( 1/\sqrt{n}\right) \end{aligned}$$

where ${\varvec{U}}_{w1i} = \int _{a_T}^{b_T} w(t)a(T_i)^{-1} [{\varvec{x}}_{i}(t) - {\varvec{e}}_{{\varvec{a}}}(t,\varvec{\beta }^0) ]dM_i(t)$ and, denoting the *i*th data point by ${\varvec{D}}_i=\{T_i,U_i,V_i,{\varvec{x}}_{i}(\cdot )\}$ and an independent data point ${\varvec{D}}_0$, ${\varvec{U}}_{w2i} = {\mathbb {E}}^*[L_i(T_0) a(T_0){\varvec{U}}_{w10}|{\varvec{D}}_i]$ and ${\varvec{U}}_{w3i} = {\mathbb {E}}^* [\int _{a_T}^{b_T} L_i^w(t) a(T_0)^{-1} [{\varvec{x}}_{0}(t) - {\varvec{e}}_{{\varvec{a}}}(t,\varvec{\beta }^0) ] dN_0(t) | {\varvec{D}}_i ] = {\varvec{0}}_p.$ Thus $\varvec{\widehat{\beta }}_w$ is asymptotically normal with plug-in variance estimator

$$\begin{aligned}&\widehat{\textrm{Var}}(\varvec{\widehat{\beta }}_w) = \nabla ^2 \varvec{\ell }(\varvec{\widehat{\beta }}_w;\varvec{\widehat{a}}, \widehat{w})^{-1} \sum _{i=1}^n \left[ \varvec{\widehat{U}}_{w1i} + \varvec{\widehat{U}}_{w2i} + \varvec{\widehat{U}}_{w3i} \right] ^{\otimes 2} \nabla ^2 \varvec{\ell }(\varvec{\widehat{\beta }}_w;\varvec{\widehat{a}}, \widehat{w})^{-1} \\&where \ \varvec{\widehat{U}}_{w1i} = \int _{a_T}^{b_T} \frac{\widehat{w}(t)}{\widehat{a}(T_i)}\left[ {\varvec{x}}_{i}(t) - \varvec{\overline{x}}_{\varvec{\widehat{a}}}(t,\varvec{\widehat{\beta }}_w)\right] d\widehat{M}_i(t),\\&\hspace{10mm}\varvec{\widehat{U}}_{w2i} = \frac{1}{n}\sum _{j=1}^n \widehat{L}_i(T_j)\widehat{a}(T_j)\varvec{\widehat{U}}_{w1j}, \\&\hspace{10mm}\varvec{\widehat{U}}_{w3i} = \frac{1}{n}\sum _{j=1}^n \widehat{L}^w_i(T_j)\widehat{a}(T_j)^{-1}\int _{a_T}^{b_T}\left[ {\varvec{x}}_{j}(t) - \varvec{\overline{x}}_{\varvec{\widehat{a}}}(t,\varvec{\widehat{\beta }}_w)\right] dN_j(t). \end{aligned}$$

The proof for Theorem 1 is provided in Appendix A.

#### Remark 3

(Computing the standard errors) The model components needed to compute the variance estimator in Theorem 1 are easy to obtain with standard software. If $\varvec{\widehat{\beta }}_w$ is obtained by the offset approach described in Sect. 2.4, then the $\varvec{\widehat{U}}_{w1i}$’s are the weighted score residuals and the $\varvec{\widehat{U}}_{w2i}$’s are weighted averages of those score residuals. The $\varvec{\widehat{U}}_{w3i}$’s are weighted averages of the Schoenfeld residuals and are asymptotically negligible when the fitted model is correctly specified. Lastly, $- \nabla ^2 \varvec{\ell }(\varvec{\widehat{\beta }}_w;\varvec{\widehat{a}}, \widehat{w})^{-1}$ is the observed inverse information matrix, which can be easily obtained from Cox regression software.

#### Remark 4

(Comparison with previous work) Mandel et al. (2018) studied the special case of stabilized weights $\widehat{w}(t)=\widehat{a}(t)$. They provided an asymptotic representation for $\varvec{\widehat{\beta }}_w-\varvec{\beta }^0$ as a U-statistic with a fairly complicated kernel of degree 3. The standard errors provided in Theorem 1, on the other hand, are more directly useful for practical implementation and cover a wide variety of weight functions $\widehat{w}(\cdot )$.

#### Remark 5

(Model misspecification and non-proportional hazards) The standard errors in Theorem 1 are still consistent under model misspecification. When the fitted model is incorrect, the IPW estimator with time-varying weights can be shown to converge to a well-defined constant $\varvec{\beta }_w^*$ under fairly general conditions (Struthers and Kalbfleisch 1986; Sasieni 1993b). The limiting value $\varvec{\beta }_w^*$ will depend on the chosen weight function $\widehat{w}(\cdot )$, and under non-proportional hazards it has an approximate interpretation as an average regression effect. In particular, survival function weighting in the case of a single binary covariate leads to a $\varvec{\beta }_w^*$ that closely approximates the log-odds of concordance between the two covariate groups, with the concordance probability defined as ${\mathbb {P}}(T_1<T_2|{\varvec{x}}_1=1, {\varvec{x}}_2=0)$ (Schemper et al. 2009). This quantity has a clear interpretation regardless of whether the proportional hazards assumption holds. Further details and related simulation results are provided in Online Resource Section S5.4.

#### Remark 6

(Sensitivity analysis for the positivity assumption) As mentioned above, the proposed standard errors are consistent under model misspecification, which may result in ${\varvec{U}}_{w3i} \ne {\varvec{0}}_p$. This is particularly useful for the sensitivity analysis estimator $\varvec{\widehat{\beta }}_{w, p_{r|l}}$, since in practice one will use multiple values for the truncated mass $p_{r|l}$, and at most one can be correct. Define $\widetilde{M}_i(t)$ by replacing $Y_i(t)$ with $\widetilde{Y}_i(t;\varvec{\widehat{\beta }}_{w,p_{r|l}},p_{r|l})$ in $\widehat{M}_i(t)$, and let $\varvec{Dr}(\varvec{\beta };\varvec{\widehat{a}}, \widehat{w})$ be the derivative of ${\varvec{r}}(\varvec{\beta };\varvec{\widehat{a}}, \widehat{w})$. In Online Resource Section S4 we show that $\varvec{\widehat{\beta }}_{w, p_{r|l}}$ is asymptotically normal with plug-in variance estimator

$$\begin{aligned}&\widehat{\textrm{Var}}(\varvec{\widehat{\beta }}_{w,p_{r|l}})\\&= \left[ \varvec{Dr}(\varvec{\widehat{\beta }}_{w,p_{r|l}};\varvec{\widehat{a}}, \widehat{w})\right] ^{-1} \sum _{i=1}^n \left[ \varvec{\widetilde{U}}_{w1i} + \varvec{\widetilde{U}}_{w2i} + \varvec{\widetilde{U}}_{w3i} \right] ^{\otimes 2} \left[ \varvec{Dr}(\varvec{\widehat{\beta }}_{w,p_{r|l}};\varvec{\widehat{a}}, \widehat{w})^{\textsf{T}}\right] ^{-1}\\&where \ \varvec{\widetilde{U}}_{w1i} = \int _{a_T}^{b_T} \frac{\widehat{w}(t)}{\widehat{a}(T_i)}\left[ {\varvec{x}}_i - \varvec{\widetilde{x}}_{\varvec{\widehat{a}}}(t,\varvec{\widehat{\beta }}_{w,p_{r|l}})\right] d\widetilde{M}_i(t),\\&\hspace{9mm}\ \varvec{\widetilde{U}}_{w2i} = \frac{1}{n}\sum _{j=1}^n \widehat{L}_i(T_j)\widehat{a}(T_j)\varvec{\widetilde{U}}_{w1j},\\&\hspace{9mm}\ \varvec{\widetilde{U}}_{w3i} = \frac{1}{n}\sum _{j=1}^n \widehat{L}^w_i(T_j)\widehat{a}(T_j)^{-1}\int _{a_T}^{b_T}\left[ {\varvec{x}}_j - \varvec{\widetilde{x}}_{\varvec{\widehat{a}}}(t,\varvec{\widehat{\beta }}_{w,p_{r|l}})\right] dN_j(t). \end{aligned}$$

These standard errors are also easily adapted to the right truncation setting, where Vakulenko-Lagun et al. (2020) instead relied on bootstrap resampling.

#### Theorem 2

(Influence function for the IPW baseline hazard estimator) Under Assumptions 1-6, suppose $\varvec{\widehat{\beta }}$ is asymptotically linear with influence function ${\varvec{L}}_i^\beta$. Then, uniformly in $t\in [a_T,\tau ]$ for $\tau <b_T$, the IPW baseline hazard estimator ${\widehat{\Lambda }}_{0}(t;\varvec{\widehat{\beta }},\varvec{\widehat{a}}) = n^{-1}\sum _{i=1}^n \int _{-\infty }^t \widehat{a}(T_i)^{-1}{\varvec{S}}_{\varvec{\widehat{a}}}^{(0)}(s,\varvec{\widehat{\beta }})^{-1}dN_i(s)$ satisfies

$$\begin{aligned} {\widehat{\Lambda }}_0(t;\varvec{\widehat{\beta }}, \varvec{\widehat{a}}) - \Lambda _0(t) = \frac{1}{n}\sum _{i=1}^n\left[ L^{\Lambda 1}_i(t) + L^{\Lambda 2}_i(t) + L^{\Lambda 3}_i(t) \right] + o_p\left( 1/\sqrt{n}\right) \end{aligned}$$

where $L^{\Lambda 1}_i(t) = - [\int _{-\infty }^t {\varvec{e}}_{{\varvec{a}}}(s,\varvec{\beta }^0)d\Lambda _0(s) ]^{\textsf{T}}{\varvec{L}}_i^\beta$ and, for ${\varvec{D}}_i=\{T_i,U_i,V_i,{\varvec{x}}_{i}(\cdot )\}$ and an independent ${\varvec{D}}_0$, $L^{\Lambda 2}_i(t) = {\mathbb {E}}^* [L_i(T_0) \int _{-\infty }^t {\varvec{s}}_{{\varvec{a}}}^{(0)}(s,\varvec{\beta }^0)^{-1}dM_0(s) | {\varvec{D}}_i ]$ and $L^{\Lambda 3}_i(t) = \int _{-\infty }^t a(T_i)^{-1}{\varvec{s}}_{{\varvec{a}}}^{(0)}(s,\varvec{\beta }^0)^{-1}dM_i(s).$ The plug-in estimates when $\varvec{\widehat{\beta }}$ is the robust IPW estimator from Theorem 1 are given by $\widehat{L}^{\Lambda 1}_i(t) = [\int _{-\infty }^t \varvec{\overline{x}}_{\varvec{\widehat{a}}}(s,\varvec{\widehat{\beta }}_w)d{\widehat{\Lambda }}_0(s;\varvec{\widehat{\beta }}_w,\varvec{\widehat{a}}) ]^{\textsf{T}} \varvec{\widehat{L}}_i^{\beta }$, where $\varvec{\widehat{L}}_i^{\beta } = [\nabla ^2 \varvec{\ell }(\varvec{\widehat{\beta }}_w;\varvec{\widehat{a}}, \widehat{w})/n ]^{-1} (\varvec{\widehat{U}}_{w1i} + \varvec{\widehat{U}}_{w2i} + \varvec{\widehat{U}}_{w3i} )$ estimates negative ${\varvec{L}}_i^\beta$, $\widehat{L}^{\Lambda 3}_i(t) = \int _{-\infty }^t \widehat{a}(T_i)^{-1}{\varvec{S}}_{\varvec{\widehat{a}}}^{(0)}(s,\varvec{\widehat{\beta }}_w)^{-1}d\widehat{M}_i(s)$, and $\widehat{L}^{\Lambda 2}_i(t) = n^{-1}\sum _{j=1}^n \widehat{L}_i(T_j)\widehat{a}(T_j) \widehat{L}^{\Lambda 3}_j(t)$.

The proof for Theorem 2 is provided in Online Resource Section S4.

## Simulation results

We assessed the accuracy of the proposed estimators and their standard errors through extensive simulations. For each setting, 1000 simulations were conducted. In each simulation we fit the standard IPW partial likelihood estimator (W-1) as well as several choices of time-varying weights, with $\widehat{w}(t)$ equal to the survival function $1-\widehat{F}(t)$ (W-surv), general Fleming-Harrington weights $\widehat{F}(t)^r[1-\widehat{F}(t)]^s$ (W-fhrs) for $(r,s)\in \{0,0.5,1,2\}\times \{0,0.5,1,2\}$, and their stabilized weighting counterparts $\widehat{a}(t)$ (W-a), $\widehat{a}(t)[1-\widehat{F}(t)]$ (W-asurv), and $\widehat{a}(t)\widehat{F}(t)^r[1-\widehat{F}(t)]^s$ (W-afhrs), using the novel methods developed in Sect. 2.2. For comparison we also included the standard maximum partial likelihood estimator, which does not account for truncation bias (UW), and the conditional maximum likelihood estimator (MLE) (Rennert and Xie 2022). Standard errors for the IPW estimators were computed using the sandwich estimator from Theorem 1, either (1) based on only $\varvec{\widehat{U}}_{w1i}$, the naive sandwich standard errors that do not account for the variability of the NPMLE, (2) based on $\varvec{\widehat{U}}_{w1i} + \varvec{\widehat{U}}_{w2i}$, the proposed standard errors trusting that the model is correct when using time-varying weights, or (3) based on $\varvec{\widehat{U}}_{w1i} + \varvec{\widehat{U}}_{w2i} + \varvec{\widehat{U}}_{w3i}$, the proposed robust variance estimator that is consistent under model misspecification. We did not compute standard errors for the MLE due to the high computational burden of bootstrapping.

**Fig. 1:**
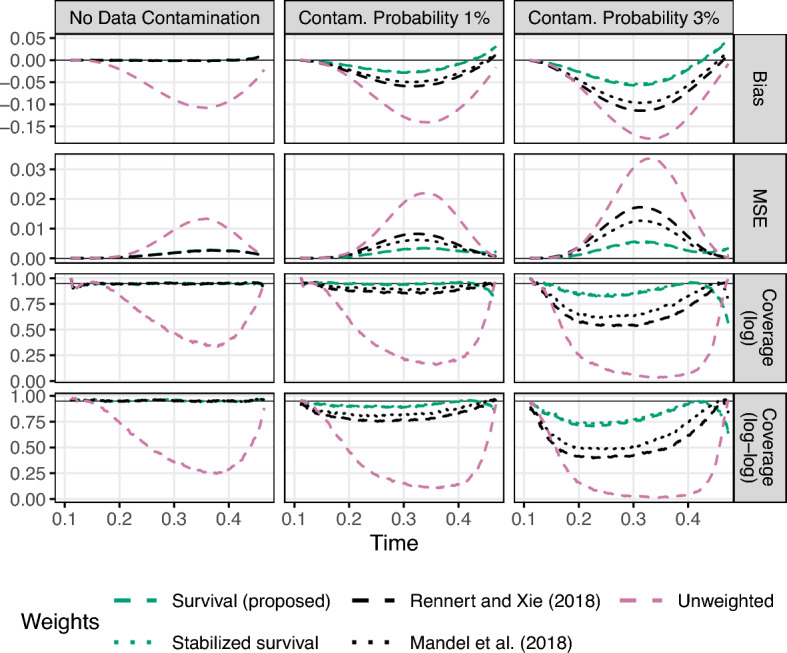
Simulation results for baseline survival function estimation with contaminated data at $n=500$. Includes the standard unweighted estimator, which does not account for double truncation bias, in pink dashed lines, the existing estimators in black lines (dashed for Rennert and Xie (2018), dotted for Mandel et al. (2018)), and the proposed estimators in green lines (dashed for $\widehat{w}(t) = 1-\widehat{F}(t)$, dotted for $\widehat{w}(t)=\widehat{a}(t)[1-\widehat{F}(t)]$). The 95% pointwise confidence intervals are computed using either a $\log$ or $\log -\log$ transformation of the baseline survival function

First, we ran simulations to check the accuracy of the proposed standard errors. The truncation times were generated as $U\sim \text {beta}(1,3)\times 0.5$ and $V = U+\text {beta}(2,10) + 0.1$. The event times followed a Cox model with hazard rate $\lambda (t|{\varvec{x}}) = \exp ({\varvec{x}}^{\textsf{T}}\varvec{\beta }^0)\lambda _0(t)$, with the baseline hazard $\lambda _0(t)$ from a $\text {beta}(2, 1.5)\times 0.4 + 0.1$ distribution, covariates ${\varvec{x}}=(x_1, x_2)^{\textsf{T}}$ with $x_1\sim$ Bernoulli(0.5) independent of $x_2\sim$ Uniform(0, 1), and $\varvec{\beta }^0=(1,1)^{\textsf{T}}$. We set the sample size to $n=500$. The results for the coefficient estimators are summarized in Table 1 (under no data contamination), including a representative subset of the Fleming-Harrington weighted estimators for easier comparisons. Here all inverse probability weighted estimators are unbiased, and their confidence intervals have the correct 95% coverage probability when using the proposed standard errors (adj. and robust). As expected, the unadjusted sandwich standard errors (unadj.) tend to underestimate the true variability of the IPW estimators. On the other hand, including $\varvec{\widehat{U}}_{w3i}$ does not have a tangible impact on the accuracy of the standard errors because the fitted model is correctly specified. Overall, we find that the MLE and all IPW estimators have roughly equivalent performance in this setting of well-behaved data, although the MLE happens to have a slightly higher variance than some IPW estimators (see Online Resource Section S5.3.2 for results at $n=300$ and 700). We also evaluated the baseline survival function estimators and their proposed standard errors in this simulation setting. As shown in Fig. 1 (under no data contamination), the IPW estimators are all unbiased, have correct confidence interval coverage rates, and are all roughly equivalent in terms of performance.

**Table 1:** Simulation results for regression coefficient estimation with contaminated data at $n=500$. The truncation rates were 0.15 (left), 0.21 (right), 0.36 (overall). Includes estimators for $\varvec{\beta }^0_1=1$ using the standard unadjusted (UW) and IPW partial likelihood (W-1), as well as time-varying weights based on the survival function $1-\widehat{F}(t)$ (W-surv), general Fleming-Harrington weights $\widehat{F}(t)^r[1-\widehat{F}(t)]^s$ (W-fhrs), and their stabilized weighting counterparts $\widehat{a}(t)$ (W-a), $\widehat{a}(t)[1-\widehat{F}(t)]$ (W-asurv), and $\widehat{a}(t)\widehat{F}(t)^r[1-\widehat{F}(t)]^s$ (W-afhrs). 95% confidence intervals (CI) are obtained from a normal approximation and either unadjusted sandwich standard errors (unadj.) or the proposed standard errors that account for the NPMLE variability (adj. and robust)

Estimator	Bias (%)	MSE	SD	Standard error			CI coverage		
					Proposed			Proposed	
				Unadj	Adj	Robust	Unadj	Adj	Robust
No data contamination									
W-1^*a*^	1.12	0.014	0.119	0.110	0.113		0.930	0.940	
W-$\hbox {surv}^{d}$	0.72	0.015	0.120	0.116	0.118	0.119	0.943	0.946	0.947
W-fh02^*c*^	0.60	0.018	0.134	0.131	0.133	0.133	0.950	0.955	0.954
W-fh11^*c*^	1.15	0.015	0.122	0.115	0.118	0.119	0.934	0.939	0.940
W-$\hbox {a}^{b}$	1.06	0.014	0.117	0.110	0.113	0.112	0.933	0.939	0.939
W-$\hbox {asurv}^{d}$	0.75	0.014	0.120	0.115	0.118	0.118	0.941	0.948	0.949
W-afh02^*c*^	0.64	0.017	0.131	0.128	0.130	0.130	0.944	0.948	0.949
W-afh11^*c*^	1.11	0.015	0.122	0.116	0.118	0.119	0.933	0.940	0.944
MLE	1.65	0.018	0.134						
UW	$-$12.98	0.027	0.100	0.096			0.730		
Contamination probability: 1%									
W-1^*a*^	$-$12.69	0.040	0.154	0.120	0.122		0.793	0.799	
W-$\hbox {surv}^{d}$	$-$6.05	0.019	0.123	0.118	0.120	0.120	0.910	0.917	0.918
W-fh02^*c*^	$-$4.06	0.020	0.135	0.131	0.133	0.133	0.930	0.934	0.935
W-fh11^*c*^	$-$9.58	0.027	0.133	0.122	0.123	0.124	0.871	0.876	0.879
W-$\hbox {a}^{b}$	$-$10.54	0.030	0.137	0.118	0.119	0.120	0.836	0.842	0.842
W-$\hbox {asurv}^{d}$	$-$5.69	0.018	0.121	0.117	0.119	0.119	0.919	0.924	0.925
W-afh02^*c*^	$-$4.05	0.019	0.131	0.128	0.131	0.130	0.930	0.935	0.937
W-afh11^*c*^	$-$8.66	0.024	0.130	0.121	0.123	0.123	0.884	0.887	0.888
MLE	$-$9.28	0.031	0.149						
UW	$-$21.59	0.059	0.110	0.101			0.419		
Contamination probability: 3%									
W-1^*a*^	$-$29.50	0.110	0.151	0.131	0.131		0.390	0.393	
W-$\hbox {surv}^{d}$	$-$15.52	0.039	0.121	0.119	0.120	0.120	0.733	0.737	0.738
W-fh02^*c*^	$-$11.28	0.030	0.131	0.131	0.133	0.132	0.862	0.865	0.864
W-fh11^*c*^	$-$23.10	0.071	0.131	0.127	0.127	0.128	0.548	0.546	0.557
W-$\hbox {a}^{b}$	$-$24.92	0.080	0.135	0.124	0.125	0.125	0.478	0.478	0.480
W-$\hbox {asurv}^{d}$	$-$14.75	0.036	0.120	0.119	0.120	0.120	0.759	0.766	0.766
W-afh02^*c*^	$-$11.24	0.029	0.129	0.129	0.130	0.130	0.857	0.859	0.860
W-afh11^*c*^	$-$21.16	0.061	0.128	0.125	0.126	0.126	0.600	0.604	0.607
MLE	$-$22.13	0.070	0.146						
UW	$-$32.54	0.117	0.105	0.101			0.125		

^*a*^ Rennert and Xie (2018)

^*b*^Mandel et al. (2018)

^*c*^ Weights motivated by the $G^{r,s}$ tests of Fleming and Harrington (2013)

^*d*^ Proposed estimators

Next, we examined robustness to data contamination. The data was contaminated by replacing a fifth of the linear predictors ${\varvec{x}}^{\textsf{T}}\varvec{\beta }^0$ that had values above a cutoff *c* with standard normal random variables, and then using these contaminated linear predictors to simulate the event times. In other words, the data followed the Cox model $\lambda (t|{\varvec{x}}) = \exp (\rho {\varvec{x}}^{\textsf{T}}\varvec{\beta }^0 + (1-\rho )z)\lambda _0(t)$ where $z\sim N(0,1)$ and $\rho \sim {\varvec{1}}({\varvec{x}}^{\textsf{T}}\varvec{\beta }^0>c)\times$Bernoulli(0.2). Thus a portion of the highest-risk individuals (according to the fitted Cox model) had long survival times that followed a different model. We ran two settings, with the cutoff *c* chosen to produce an overall contamination probability of 0.01 and 0.03, respectively, motivated by a similar simulation study for weighted Cox regression under right censoring (Sasieni 1993a). Again the sample size was set to $n=500$. We also explored sample sizes of $n=300$, which is similar to the AIDS dataset in Sect. 5, and $n=700$ (see Online Resource Section S5.3.2).

The results for the coefficient estimators under data contamination are summarized in Table 1. Although all estimators have increased bias as the contamination percentage increases, using the proposed time-varying weights based on (powers of) the survival function clearly leads to the lowest bias, lowest MSE, and most accurate confidence intervals out of all estimators by a wide margin. In particular, our proposed estimators (W-surv and W-asurv) attain 60% of the MSE of the existing estimators (Rennert and Xie 2018; Mandel et al. 2018; Rennert and Xie 2022), which do not have time-varying weights based on the event time distribution, and 80% of the MSE of Fleming-Harrington weights with $r=s=1$, which use the NPMLE $\widehat{F}(t)$ to downweight both early and late event times. Whether one uses the survival function alone (W-surv) or its stabilized counterpart (W-asurv) makes relatively little difference. The same trends occur at other sample sizes as well (Online Resource Section S5.3.2). Similarly, the proposed survival function weights produce the most robust estimator of the baseline survival function, as shown in Fig. 1. Again, they result in lower bias, lower MSE, and more accurate confidence interval coverage than the weights used by both existing estimators. Since the estimators based on Fleming-Harrington weights performed similar to Mandel et al. (2018) weights (for $r=s=1$) and survival function weights (for $r=0,s=2$), we omit their results for clarity.

We observed several notable trends in the simulation results for the full set of Fleming-Harrington weighted estimators with $\widehat{w}(t) = \widehat{F}(t)^r[1-\widehat{F}(t)]^s, (r,s)\in \{0,0.5,1,2\}\times \{0,0.5,1,2\}$ (some not reported in Table 1). First, weights constructed from only the CDF ($r>0,s=0$) generally had the worst bias and variance across all settings, so we chose not to report their results. This may be explained by the fact that such weights emphasize long event times, which makes the estimator more sensitive to influential points. This would also explain why weights constructed from only the survival function ($r=0,s>0$) typically had the best MSE across all settings. Among this class of weights, choosing the best power *s* involved a bias-variance tradeoff, where larger *s* improved robustness under data contamination while also increasing the variance of the estimator. The best choice may depend on the distribution of the data and thus be difficult to determine in practice. Therefore, we recommend the proposed survival function weights $(r=0,s=1)$ as a generally reasonable choice with much better robustness than the existing double truncation methods.

Finally, we simulated data under violations of the positivity assumption. The event times followed the same Cox model as in Table 1 (without data contamination), which had support [0.1, 0.5]. The uniform covariate was centered for better interpretability of the baseline distribution. The truncation times were $U\sim \text {Uniform}(0.18, 0.22)$ and $V=U+\text {Uniform}(0.05, 0.09)$, which resulted in a true truncated mass of $p^*_{r|l}=4/9$. We fit sensitivity estimators $\varvec{\widehat{\beta }}_{w,p_{r|l}}$ for values of $p_{r|l}$ ranging from 0 to 1, in increments of 0.05, using the novel method outlined in Sect. 2.3 and the standard errors developed in Sect. 3.2.

Table 2 summarizes the results for a subset of the fitted sensitivity estimators at $n=500$. First, we note that the IPW estimators which do not consider potential positivity violations (with $p_{r|l}=0$) have a heavy bias of about $-60\%$ and 95% confidence interval coverage rates of less than $40\%$. Thus all existing methods would heavily underestimate the true hazard ratio. In contrast, the sensitivity analysis estimators near the true truncated mass are approximately unbiased, and their sensitivity intervals have roughly 95% coverage. As long as the truncated mass is not underestimated, all sensitivity intervals achieve at least 95% coverage. The two sensitivity analysis estimators that use the proposed survival function weights (W-surv and W-asurv) generally have the lowest variance and thinnest sensitivity intervals, leading to higher power compared to the novel sensitivity analysis estimators that use weights adapted from the existing methods of Rennert and Xie (2018) (W-1) and Mandel et al. (2018) (W-a).

**Table 2:** Simulation results for proposed sensitivity analysis under violated positivity assumption at $n=500$. Includes novel IPW sensitivity analysis estimators with weights adapted from the standard IPW partial likelihood estimator (W-1), the proposed time-varying weights based on the survival function $1-\widehat{F}(t)$ (W-surv), and their stabilized weighting counterparts $\widehat{a}(t)$ (W-a) and $\widehat{a}(t)[1-\widehat{F}(t)]$ (W-asurv). The true truncated mass was 0.1 on the left and 0.4 on the right, i.e. $p_{r|l}=0.44$, while the true coefficient value was $\varvec{\beta }^0_1=1$. The sensitivity intervals (SI) have nominal level 0.95 when the truncated mass is not underestimated

Quantity	Estimator	Assumed truncated mass $p_{r|l}$									
		0.00	0.10	0.20	0.30	0.40	0.45	0.50	0.60	0.70	0.80
Bias of $\varvec{\widehat{\beta }}_{w,p_{r|l}}$	W-1	$-$0.62	$-$0.39	$-$0.27	$-$0.16	$-$0.05	0.02	0.08	0.24	0.44	0.71
	W-surv	$-$0.56	$-$0.38	$-$0.27	$-$0.16	$-$0.05	0.01	0.07	0.23	0.41	0.68
	W-a	$-$0.60	$-$0.39	$-$0.28	$-$0.16	$-$0.04	0.04	0.10	0.26	0.45	0.72
	W-asurv	$-$0.57	$-$0.39	$-$0.29	$-$0.17	$-$0.05	0.01	0.07	0.23	0.42	0.68
SD	W-1	0.37	0.47	0.49	0.55	0.60	0.63	0.67	0.76	0.84	0.98
	W-surv	0.31	0.41	0.45	0.51	0.57	0.61	0.65	0.71	0.81	0.95
	W-a	0.30	0.40	0.50	0.56	0.63	0.65	0.70	0.79	0.92	1.11
	W-asurv	0.27	0.36	0.43	0.49	0.53	0.57	0.62	0.67	0.76	0.92
SI length	W-1	0.96	1.34	1.54	1.75	1.97	2.08	2.21	2.52	2.90	3.42
	W-surv	0.92	1.24	1.45	1.65	1.88	1.99	2.12	2.43	2.81	3.33
	W-a	0.87	1.25	1.50	1.75	2.00	2.14	2.30	2.65	3.07	3.66
	W-asurv	0.80	1.11	1.31	1.53	1.75	1.88	2.01	2.34	2.75	3.29
SI coverage	W-1	0.35	0.72	0.85	0.90	0.93	0.94	0.95	0.97	0.97	0.98
	W-surv	0.36	0.73	0.88	0.93	0.95	0.96	0.97	0.98	0.99	0.99
	W-a	0.30	0.72	0.86	0.92	0.95	0.95	0.96	0.98	0.99	0.98
	W-asurv	0.28	0.64	0.83	0.90	0.95	0.96	0.97	0.98	0.99	0.99

## Real data analysis

We applied the proposed estimators to analyze the relationship between age and AIDS incubation time from transfusion-acquired HIV. The data was collected by the Centers for Disease Control (CDC) and is publicly available in the R package gss (Gu 2014, v2.2-7). The patients were sampled retrospectively, conditional on AIDS diagnosis between its discovery in 1982 and the end of the study in 1986. Time was measured in months since HIV infection, so the truncation times $U_i=\{\text {time from ith patient's HIV infection to 1982}\}$ and $V_i=\{\text {time from ith patient's HIV infection to 1986}\}$ varied across individuals. The single covariate was age at infection, with three groups: children (age $\le 4$, $n=34$), adults (age 5-59, $n=120$), and elderly (age $>59$, $n=141$). The reference group was elderly patients. We did not find strong evidence against quasi-independent truncation (Assumption 3; see Sect. 5.1), and we found that the data satisfied the strong connectedness condition for NPMLE identifiability (Assumption 2) using the is.connected function from the R package igraph (Csárdi and Nepusz 2006; Csárdi et al. 2025, v1.5.0). Since we observed that the NPMLE $\widehat{a}(T_i)$ approached zero for patients with longer incubation times, we chose to analyze this data using the stabilized survival function time weight $\widehat{w}(t) = \widehat{a}(t)[1-\widehat{F}(t)]$ to allow robustness against extreme inverse probability weights.

Existing literature has estimated the median incubation time of AIDS from transfusion-acquired HIV infection to be around 20 months for children, 90 months for adults, and 65 months for the elderly (Medley et al. 1987; Blaxhult et al. 1990; Kopec-Schrader et al. 1993). For comparison, we computed the NPMLE of the incubation time distribution for each of the three age groups. The estimated median (range) was 18 (4-43) months for children, 63 (4-89) months for adults, and 64 (0.5-83) months for the elderly age group. Evidently there is likely a severe positivity violation in this dataset, with possibly 50% of the probability mass for the adult incubation time distribution being right-truncated.

**Table 3:** Regression coefficient estimates and 95% sensitivity intervals (SI) for AIDS incubation data, under several assumed values for the truncated mass $p_{r|l}$. The single categorical covariate was age group, with reference group age $>59$. Here $\varvec{\widehat{\beta }}$ is the coefficient estimate for the given age group

$p_{r|l}$	Age $\le 4$			Age 5-59		
	${\widehat{\beta }}$	Lower SI	Upper SI	${\widehat{\beta }}$	Lower SI	Upper SI
0.00	2.14	1.47	2.81	$-$0.69	$-$1.77	0.38
0.02	2.16	1.47	2.83	$-$0.94	$-$2.88	1.01
0.04	2.18	1.47	2.85	$-$1.10	$-$3.69	1.48
0.06	2.20	1.47	2.87	$-$1.28	$-$4.66	2.10
0.08	2.22	1.47	2.89	$-$1.48	$-$5.95	3.00
0.10	2.24	1.47	2.91	$-$1.73	$-$7.87	4.42
0.12	2.26	1.47	2.93	$-$2.05	$-$11.14	7.04
0.14	2.29	1.47	2.96	$-$2.54	$-$18.36	13.28

The results of the data analysis are provided in Table 3. Note that, relative to elderly patients, children are estimated to have shorter incubation times on average, while adults are estimated to have longer incubation times. This is intuitive and also supported by prior clinical literature (Medley et al. 1987; Blaxhult et al. 1990; Kopec-Schrader et al. 1993), since adults are expected to have the most robust immune systems among the three age groups. When we assumed there was no truncated mass ($p_{r|l}=0$), the estimated hazard ratios were 8.5 for children and 0.5 for adults, relative to the elderly, but the latter regression effect was not statistically significant. Given the positivity issues outlined above, we also conducted a sensitivity analysis to assess the robustness of our Cox regression estimates. The results, also provided in Table 3, indicate that we are potentially severely underestimating the magnitude of the regression effect for adults. The estimates for children, on the other hand, are fairly stable. This is in-line with the fact that adults tend to have the longest incubation times, so they are more likely to be right-truncated due to non-positivity compared to the other age groups.

### Assessing the quasi-independent truncation assumption

To check for potential violations of the quasi-independent truncation assumption, we applied the proposed nonparametric diagnostic described in Sect. 2.1, stratified by age group. First, we checked the quasi-independent truncation assumption within strata. The estimated conditional Kendall’s tau rank correlation between the incubation times and truncation times was 0.06 for children, 0.04 for adults, and 0.12 among the elderly. The aggregate p-value for testing whether all three rank correlations were zero was 0.05. Given the small effect sizes and marginally significant p-value, we did not consider this strong evidence against the quasi-independent truncation assumption within each strata.

**Fig. 2:**
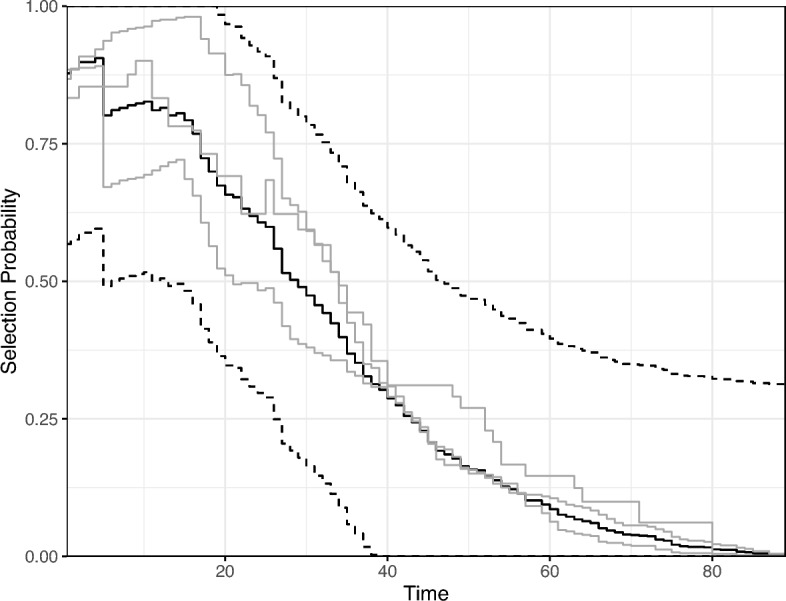
Diagnostic plot for assessing the quasi-independent truncation assumption in the AIDS data, stratified by age group. Includes the stratum-specific NPMLE’s ${\widehat{\pi }}(t|s)$ (gray lines), the unstratified NPMLE ${\widehat{\pi }}(t)$ (solid black line), and a 95% uniform confidence band for ${\widehat{\pi }}(t|s) - {\widehat{\pi }}(t)$ centered at ${\widehat{\pi }}(t)$ (black dashed lines)

Then, we checked the quasi-independent truncation assumption across strata. The diagnostic plot in Fig. 2 includes a 95% uniform confidence band which shows no significant difference between the stratum-specific and unstratified selection probabilities. The p-value for testing the null hypothesis that $\sup _{t\in [a_T,b_T],s} |\pi (t|s) - \pi (t)|=0$ was 0.2. The confidence band and p-value were computed based on 2000 simulations from the estimated asymptotic null distribution of $\sup _{t\in [a_T,b_T],s} |{\widehat{\pi }}(t|s) - {\widehat{\pi }}(t)|$. In conclusion, we did not find strong evidence that the quasi-independent truncation assumption was violated in this dataset.

## Discussion

In this paper, we have proposed robust IPW estimation for doubly truncated Cox regression with time-varying weights based on the survival function, which has been shown to be highly robust against influential points with contaminated data. In particular, our simulation results have shown that the proposed estimators can achieve lower bias and MSE than the current IPW estimators of Rennert and Xie (2018) and Mandel et al. (2018) in such settings. Although the estimator of Mandel et al. (2018) can be thought of as a robust IPW estimator with time-varying weights based on the normalized sampling probabilities $\widehat{w}(t)=\widehat{a}(t)$, our proposed estimators consistently showed better robustness properties across several simulation settings. This may be explained by the fact that the sampling probability is determined solely by the bivariate truncation time distribution, which does not allow their stabilized weights to automatically adapt to different event time distributions. We have further derived closed-form standard errors for a general class of IPW estimators based on NPMLE weights, which includes existing methods as well as our robust alternatives. Lastly, we have developed a graphical diagnostic and statistical test for the quasi-independent truncation assumption when it involves covariates, as well as sensitivity analysis approaches regarding the positivity assumption on the sampling probabilities. One possible direction for future work would be to relax the quasi-independent truncation assumption to covariate-dependent truncation.

Our proposed diagnostic for quasi-independent truncation is based on stratifying by binned covariate values, which leads to a relatively simple testing procedure. For each stratum, however, neither the within-stratum quasi-independence test nor the within-stratum selection probability estimates ${\widehat{\pi }}(t|s)$ use any information from the other strata. This may limit the power of the test when there are a large number of covariates, particularly if many of them are continuous. To mitigate this issue, the within-strata quasi-independence tests could be replaced by a modification of the conditional Kendall’s tau test (Martin and Betensky 2005) with kernel smoothing across covariate values. Estimating the conditional selection probabilities ${\widehat{\pi }}(t|{\varvec{x}})$ nonparametrically without stratification, on the other hand, would require a novel estimator that is beyond the scope of this paper. Other approaches to (conditional) independence testing have been studied for untruncated samples (Su and White 2014; Shah and Peters 2020; Zhou et al. 2020; Cai et al. 2022), but their extension to doubly truncated data is complicated by the sampling bias.

Our results have provided useful tools for the analysis of observational studies subject to double truncation. Through our developments for the NPMLE and our robust IPW estimators, we provide fast extensions of standard methods for right censored data, where inference is based on Kaplan-Meier curves and the maximum partial likelihood estimator, to doubly truncated data. Testing for ignorable sampling bias through the NPMLE is easy to implement and computationally fast. Also, using our closed-form standard errors for Cox regression will expedite the model building process and facilitate sensitivity analysis for the positivity assumption without the unnecessary computational burden of resampling methods. The R code for the data analysis in Sect. 5 is available online at https://github.com/omar-vazquez/robust_ipw.

In practice, the analyst can apply standard weighted residual diagnostics with our proposed IPW estimators to detect model misspecification and update the model accordingly. To address non-proportional hazards, our proposed estimators can be easily extend to fit stratified Cox models. Another option is to add new time-varying covariates to the model based on interactions between existing covariates and functions of time. Unfortunately, this will complicate the interpretation of the coefficient estimates, since the log hazard ratios now vary over time. A third option would be to neither stratify nor add these interactions, and just fit the misspecified Cox model. This can provide a simple and useful summary of the covariate effects, since existing work implies that the coefficient estimates from an IPW estimator with time-varying weights based on the weight function *w*(*t*) will approximate a weighted average of the true log hazard ratios over time, with weights $\propto w(t)$ (see Remark 5 and Supplementary material S5.4). The proposed survival function weights are an intuitive choice in this context, since each time point is weighted by the proportion of individuals still at-risk.

Using our proposed diagnostic, we found that the quasi-independent truncation assumption was satisfied in the AIDS data to a reasonable extent. Nevertheless, we did observe a small positive Kendall’s tau correlation between the incubation and truncation times in Sect. 5.1, mostly in the elderly age group. This could be caused by a delay in diagnosis time for patients infected far before AIDS discovery. In that case, the reported AIDS diagnosis times would overestimate the true incubation times resulting in measurement error for the event times. Accounting for this potential issue goes beyond correcting for double truncation bias and may require some additional modeling.

## Supplementary Information

Below is the link to the electronic supplementary material.

Supplementary file 1 (pdf 457 KB)

## Appendix A Proof of Theorem 1

By a first order Taylor series expansion of the score function, we have

$$\begin{aligned} {\varvec{0}}_p = \nabla \varvec{\ell }(\varvec{\widehat{\beta }}_w;\varvec{\widehat{a}}, \widehat{w}) = \nabla \varvec{\ell }(\varvec{\beta }^0;\varvec{\widehat{a}}, \widehat{w}) + \nabla ^2 \varvec{\ell }(\varvec{\tilde{\beta }};\varvec{\widehat{a}}, \widehat{w})(\varvec{\widehat{\beta }}_w - \varvec{\beta }^0) \end{aligned}$$

where $\varvec{\tilde{\beta }}$ lies on the line between $\varvec{\widehat{\beta }}_w$ and $\varvec{\beta }^0$. Therefore

$$\begin{aligned} \varvec{\widehat{\beta }}_w - \varvec{\beta }^0 = -\left[ \nabla ^2 \varvec{\ell }(\varvec{\tilde{\beta }};\varvec{\widehat{a}}, \widehat{w})/n\right] ^{-1}\frac{1}{n}\nabla \varvec{\ell }(\varvec{\beta }^0;\varvec{\widehat{a}}, \widehat{w}). \end{aligned}$$

First, by the convergence of $\varvec{\widehat{\beta }}_w$ to $\varvec{\beta }^0$ and uniform convergence of ${\varvec{S}}_{\varvec{\widehat{a}}}^{(r)}(t,\varvec{\beta }^0)$ to ${\varvec{s}}_{{\varvec{a}}}^{(r)}(t,\varvec{\beta }^0)$, $r=0,1,2$, we have

$$\begin{aligned} \frac{1}{n}\nabla ^2 \varvec{\ell }(\varvec{\tilde{\beta }};\varvec{\widehat{a}}, \widehat{w})&= \frac{1}{n} \sum _{i=1}^n \int _{a_T}^{b_T} \frac{\widehat{w}(t)}{\widehat{a}(T_i)}\left[ \varvec{\overline{x}}_{\varvec{\widehat{a}}}(t,\varvec{\beta }^0)^{\otimes 2} - \frac{{\varvec{S}}_{\varvec{\widehat{a}}}^{(2)}(t,\varvec{\beta }^0)}{{\varvec{S}}_{\varvec{\widehat{a}}}^{(0)}(t,\varvec{\beta }^0)}\right] dN_i(t) + o_p\left( 1\right) \\&= \frac{1}{n} \sum _{i=1}^n \int _{a_T}^{b_T} \frac{w(t)}{a(T_i)}\left[ {\varvec{e}}_{{\varvec{a}}}(t,\varvec{\beta }^0)^{\otimes 2} - \frac{{\varvec{s}}_{{\varvec{a}}}^{(2)}(t,\varvec{\beta }^0)}{{\varvec{s}}_{{\varvec{a}}}^{(0)}(t,\varvec{\beta }^0)}\right] dN_i(t) + o_p\left( 1\right) \end{aligned}$$

and this sum of iid random matrices converges in probability to the full rank matrix

$$\begin{aligned} -\varvec{\mathcal {I}}_w = {\mathbb {E}}\left[ \int _{a_T}^{b_T} w(t)\left\{ {\varvec{e}}_{{\varvec{a}}}(t,\varvec{\beta }^0)^{\otimes 2} - \frac{{\varvec{s}}_{{\varvec{a}}}^{(2)}(t,\varvec{\beta }^0)}{{\varvec{s}}_{{\varvec{a}}}^{(0)}(t,\varvec{\beta }^0)}\right\} dN(t) \right] \end{aligned}$$

by the law of large numbers.

We move on to the gradient. Let $\Lambda _i(t) = N_i(t) - M_i(t)$ and note that $\sum _{i=1}^n\int _{a_T}^{b_T} \widehat{w}(t)\widehat{a}(T_i)^{-1}\left[ {\varvec{x}}_{i}(t) - \varvec{\overline{x}}_{\varvec{\widehat{a}}}(t,\varvec{\beta }^0)\right] d\Lambda _i(t)={\varvec{0}}_p$. Therefore the gradient can be written as

$$\begin{aligned} \frac{1}{n}\nabla \varvec{\ell }(\varvec{\beta }^0;\varvec{\widehat{a}}, \widehat{w})&= \frac{1}{n}\sum _{i=1}^n\int _{a_T}^{b_T} \frac{\widehat{w}(t)}{\widehat{a}(T_i)}\left[ {\varvec{x}}_{i}(t) - \varvec{\overline{x}}_{\varvec{\widehat{a}}}(t,\varvec{\beta }^0)\right] dM_i(t) \\&= \underbrace{\frac{1}{n} \sum _{i=1}^n \int _{a_T}^{b_T} \frac{w(t)}{a(T_i)}\left[ {\varvec{x}}_{i}(t) - \varvec{\overline{x}}_{\varvec{\widehat{a}}}(t,\varvec{\beta }^0)\right] dM_i(t)}_{S_1} \\&\hspace{3mm} + \underbrace{\frac{1}{n} \sum _{i=1}^n \int _{a_T}^{b_T} \widehat{w}(t)\left[ \frac{1}{\widehat{a}(T_i)} - \frac{1}{a(T_i)}\right] \left[ {\varvec{x}}_{i}(t) - \varvec{\overline{x}}_{\varvec{\widehat{a}}}(t,\varvec{\beta }^0)\right] dM_i(t)}_{S_2} \\&\hspace{3mm} + \underbrace{\frac{1}{n} \sum _{i=1}^n \int _{a_T}^{b_T} \frac{\widehat{w}(t) - w(t)}{a(T_i)} \left[ {\varvec{x}}_{i}(t) - \varvec{\overline{x}}_{\varvec{\widehat{a}}}(t,\varvec{\beta }^0)\right] dM_i(t)}_{S_3}. \end{aligned}$$

For the first term $S_1$, by construction the process $n^{-1/2}\sum _{i=1}^n a(T_i)^{-1}M_i(t)$ is a sum of iid terms with uniformly mean zero. By the monotonicity of its components, it converges weakly to a mean-zero Gaussian process, so the uniform convergence of $\varvec{\overline{x}}_{\varvec{\widehat{a}}}(t,\varvec{\beta }^0)$ implies that

$$\begin{aligned} S_1&=\underbrace{\frac{1}{n} \sum _{i=1}^n \int _{a_T}^{b_T} \frac{w(t)}{a(T_i)}\left[ {\varvec{e}}_{{\varvec{a}}}(t,\varvec{\beta }^0) - \varvec{\overline{x}}_{\varvec{\widehat{a}}}(t,\varvec{\beta }^0)\right] dM_i(t)}_{o_p\left( 1/\sqrt{n}\right) } \\&\hspace{3mm} + \frac{1}{n} \sum _{i=1}^n \underbrace{\int _{a_T}^{b_T} \frac{w(t)}{a(T_i)}\left[ {\varvec{x}}_{i}(t) - {\varvec{e}}_{{\varvec{a}}}(t,\varvec{\beta }^0)\right] dM_i(t)}_{{\varvec{U}}_{w1i}} \\&= \frac{1}{n(n-1)}\sum _{i\ne j} {\varvec{U}}_{w1i} + o_p\left( 1/\sqrt{n}\right) . \end{aligned}$$

For the second term $S_2$, we first apply the uniform convergence of $\varvec{\overline{x}}_{\varvec{\widehat{a}}}(t,\varvec{\beta }^0)$, and then plug-in $\widehat{a}(t)^{-1}-a(t)^{-1}=n^{-1}\sum _jL_j(t)+o_p\left( 1/\sqrt{n}\right)$ to get

$$\begin{aligned} S_2&= \frac{1}{n} \sum _{i=1}^n \int _{a_T}^{b_T} w(t)\left[ \frac{1}{\widehat{a}(T_i)} - \frac{1}{a(T_i)}\right] \left[ {\varvec{x}}_{i}(t) - {\varvec{e}}_{{\varvec{a}}}(t,\varvec{\beta }^0)\right] dM_i(t) + o_p\left( 1/\sqrt{n}\right) \\&= \frac{1}{n^2}\sum _{i=1}^n \sum _{j=1}^n L_j(T_i)\int _{a_T}^{b_T} w(t)\left[ {\varvec{x}}_{i}(t) - {\varvec{e}}_{{\varvec{a}}}(t,\varvec{\beta }^0)\right] dM_i(t) + o_p\left( 1/\sqrt{n}\right) \\&= \frac{1}{n(n-1)}\sum _{i\ne j} L_j(T_i) a(T_i){\varvec{U}}_{w1i} + o_p\left( 1/\sqrt{n}\right) . \end{aligned}$$

Similarly, for the third term $S_3$ we apply the uniform convergence of $\varvec{\overline{x}}_{\varvec{\widehat{a}}}(t,\varvec{\beta }^0)$ to get

$$\begin{aligned} S_3&= \frac{1}{n} \sum _{i=1}^n \int _{a_T}^{b_T} \frac{\widehat{w}(t) - w(t)}{a(T_i)} \left[ {\varvec{x}}_{i}(t) - \varvec{\overline{x}}_{{\varvec{a}}}(t,\varvec{\beta }^0)\right] dM_i(t) + o_p\left( 1/\sqrt{n}\right) \\&= \frac{1}{n} \sum _{i=1}^n \int _{a_T}^{b_T} \frac{\widehat{w}(t) - w(t)}{a(T_i)} \left[ {\varvec{x}}_{i}(t) - \varvec{\overline{x}}_{{\varvec{a}}}(t,\varvec{\beta }^0)\right] dN_i(t) + o_p\left( 1/\sqrt{n}\right) , \end{aligned}$$

since $\sum _{i=1}^n\int _{a_T}^{b_T} [\widehat{w}(t) - w(t)]a(T_i)^{-1}\left[ {\varvec{x}}_{i}(t) - \varvec{\overline{x}}_{{\varvec{a}}}(t,\varvec{\beta }^0)\right] d\Lambda _i(t)={\varvec{0}}_p$, and then plug-in $\widehat{w}(t)-w(t)=n^{-1}\sum _j L_j^w(t)+o_p\left( 1/\sqrt{n}\right)$ to get

$$\begin{aligned} S_3&= \frac{1}{n^2} \sum _{i=1}^n\sum _{j=1}^n \int _{a_T}^{b_T} \frac{L_j^w(t)}{a(T_i)} \left[ {\varvec{x}}_{i}(t) - {\varvec{e}}_{{\varvec{a}}}(t,\varvec{\beta }^0)\right] dN_i(t) + o_p\left( 1/\sqrt{n}\right) \\&= \frac{1}{n(n-1)} \sum _{i\ne j} \int _{a_T}^{b_T} \frac{L_j^w(t)}{a(T_i)} \left[ {\varvec{x}}_{i}(t) - {\varvec{e}}_{{\varvec{a}}}(t,\varvec{\beta }^0)\right] dN_i(t) + o_p\left( 1/\sqrt{n}\right) . \end{aligned}$$

Thus $n^{-1}\nabla \varvec{\ell }(\varvec{\beta }^0;\varvec{\widehat{a}}, \widehat{w})=S_1+S_2+S_3$ can be written in the form of a mean zero U-statistic

$$\begin{aligned} \frac{1}{n}\nabla \varvec{\ell }(\varvec{\beta }^0;\varvec{\widehat{a}}, \widehat{w}) = \frac{1}{n(n-1)}\sum _{i<j} {\varvec{k}}({\varvec{D}}_i, {\varvec{D}}_j) + o_p\left( 1/\sqrt{n}\right) , \end{aligned}$$

with kernel

$$\begin{aligned} {\varvec{k}}({\varvec{D}}_i, {\varvec{D}}_j) = {\varvec{k}}_1({\varvec{D}}_i, {\varvec{D}}_j) + {\varvec{k}}_1({\varvec{D}}_j, {\varvec{D}}_i), \\ {\varvec{k}}_1({\varvec{D}}_i, {\varvec{D}}_j) = {\varvec{U}}_{w1i} + L_j(T_i) a(T_i){\varvec{U}}_{w1i} + \int _{a_T}^{b_T} \frac{L_j^w(t)}{a(T_i)} \left[ {\varvec{x}}_{i}(t) - {\varvec{e}}_{{\varvec{a}}}(t,\varvec{\beta }^0)\right] dN_i(t). \end{aligned}$$

Since $L_i(t)$ and $L_i^w(t)$ have uniformly mean zero, we have ${\mathbb {E}}^*[{\varvec{k}}_1({\varvec{D}}_i, {\varvec{D}}_j)|{\varvec{D}}_i]={\varvec{U}}_{w1i}$, which simplifies the form of the kernel’s Hájek projection. Thus,

$$\begin{aligned} \frac{1}{n}\nabla \varvec{\ell }(\varvec{\beta }^0;\varvec{\widehat{a}}, \widehat{w}) = \frac{1}{n}\sum _{i=1}^n {\mathbb {E}}^*[{\varvec{k}}({\varvec{D}}_i, {\varvec{D}}_0)|\varvec{D_i}] + o_p\left( 1/\sqrt{n}\right) \end{aligned}$$

with

$$\begin{aligned} {\mathbb {E}}^*[{\varvec{k}}({\varvec{D}}_i, {\varvec{D}}_0)|\varvec{D_i}] = {\varvec{U}}_{w1i} + {\varvec{U}}_{w2i} + {\varvec{U}}_{w3i}. \end{aligned}$$

This completes the proof of Theorem 1.

## References

[CR1] Binder DA (1992) Fitting Cox’s proportional hazards models from survey data. Biometrika 79(1):139–147

[CR2] Blaxhult A, Granath F, Lidman K, Giesecke J (1990) The influence of age on the latency period to aids in people infected by hiv through blood transfusion. AIDS 4(2):125–130 2328095 10.1097/00002030-199002000-00005

[CR3] Cai Z, Li R, Zhang Y (2022) A distribution free conditional independence test with applications to causal discovery. J Mach Learn Res 23(85):1–41

[CR4] Csárdi G, Nepusz T (2006) The igraph software package for complex network research. Inter J Complex Syst. 1695, https://igraph.org

[CR5] Csárdi G, Nepusz T, Traag V, Horvát S, Zanini F, Noom D, Müller K (2025) igraph: Network analysis and visualization in R. 10.5281/zenodo.7682609, https://CRAN.R-project.org/package=igraph, R package version 1.5.0

[CR6] Dörre A, Emura T (2019) Analysis of doubly truncated data: an introduction. Springer, New York

[CR7] Efron B, Petrosian V (1999) Nonparametric methods for doubly truncated data. J Am Stat Assoc 94(447):824–834

[CR8] Emura T, Konno Y, Michimae H (2015) Statistical inference based on the nonparametric maximum likelihood estimator under double-truncation. Lifetime Data Anal 21:397–418 25001399 10.1007/s10985-014-9297-5

[CR9] Fleming TR, Harrington DP (2013) Counting processes and survival analysis, vol 625. John Wiley & Sons, Hoboken

[CR10] Grambsch PM, Therneau TM (1994) Proportional hazards tests and diagnostics based on weighted residuals. Biometrika 81(3):515–526

[CR11] Gu C (2014) Smoothing spline ANOVA models: R package gss. J Stat Softw 58(5):1–25

[CR12] Kopec-Schrader E, Tindall B, Learmontt J, Wyliet B, Kaldor JM (1993) Development of aids in people with transfusion-acquired hiv infection. AIDS 7(7):1009–1014 8357547 10.1097/00002030-199307000-00016

[CR13] Lin D (2000) On fitting Cox’s proportional hazards models to survey data. Biometrika 87(1):37–47

[CR14] Mandel M, de Uña-Álvarez J, Simon DK, Betensky RA (2018) Inverse probability weighted Cox regression for doubly truncated data. Biometrics 74(2):481–487 28886206 10.1111/biom.12771 PMC5843502

[CR15] Martin EC, Betensky RA (2005) Testing quasi-independence of failure and truncation times via conditional Kendall’s tau. J Am Stat Assoc 100(470):484–492

[CR16] Medley G, Anderson R, Cox D, Billard L (1987) Incubation period of aids in patients infected via blood transfusion. Nature 328(6132):719–721 3614379 10.1038/328719a0

[CR17] Pan Q, Schaubel DE (2008) Proportional hazards models based on biased samples and estimated selection probabilities. Can J Stat 36(1):111–127

[CR18] R Core Team (2023) R: A language and environment for statistical computing. R Foundation for Statistical Computing, Vienna, Austria, https://www.R-project.org/

[CR19] Rennert L, Xie SX (2018) Cox regression model with doubly truncated data. Biometrics 74(2):725–733 29073330 10.1111/biom.12809 PMC5920791

[CR20] Rennert L, Xie SX (2022) Cox regression model under dependent truncation. Biometrics 78(2):460–473, the unofficial version of this Biometrics paper describing the same method was deposited in a preprint server in 2018 10.1111/biom.13451 PMC8426413 33687064

[CR21] Sasieni P (1993) Maximum weighted partial likelihood estimators for the Cox model. J Am Stat Assoc 88(421):144–152

[CR22] Sasieni P (1993) Some new estimators for Cox regression. Ann Stat 21(4):1721–1759

[CR23] Schemper M (1992) Cox analysis of survival data with non-proportional hazard functions. J R Stat Soc Ser D: Stat 41(4):455–465

[CR24] Schemper M, Wakounig S, Heinze G (2009) The estimation of average hazard ratios by weighted Cox regression. Stat Med 28(19):2473–2489 19472308 10.1002/sim.3623

[CR25] Shah RD, Peters J (2020) The hardness of conditional independence testing and the generalised covariance measure. Ann Stat 48(3):1514–1538

[CR26] Shen PS (2010) Nonparametric analysis of doubly truncated data. Ann Inst Stat Math 62(5):835–853

[CR27] Shen PS (2011) Testing quasi-independence for doubly truncated data. J Nonparametric Stat 23(3):753–761

[CR28] Shen PS (2025) Cox regression model with doubly truncated and interval-censored data. Comput Stat & Data Anal 203:108090

[CR29] Shen PS, Hsu H (2020) Conditional maximum likelihood estimation for semiparametric transformation models with doubly truncated data. Comput Stat & Data Anal 144:106862

[CR30] Struthers CA, Kalbfleisch JD (1986) Misspecified proportional hazard models. Biometrika 73(2):363–369

[CR31] Su L, White H (2014) Testing conditional independence via empirical likelihood. J Econom 182(1):27–44

[CR32] Therneau TM (2023) A package for survival analysis in R. https://CRAN.R-project.org/package=survival, R package version 3.5-5

[CR33] Therneau TM, Grambsch PM (2000) Modeling survival data: extending the Cox model. Springer, New York

[CR34] de Uña-Álvarez J, Keilegom IV (2021) Efron–Petrosian integrals for doubly truncated data with covariates: an asymptotic analysis. Bernoulli 27(1):249–273

[CR35] de Uña-Álvarez J (2023) Testing for an ignorable sampling bias under random double truncation. Stat Med 42(20):3732–3744 37312237 10.1002/sim.9828

[CR36] Vakulenko-Lagun B, Mandel M, Betensky RA (2020) Inverse probability weighting methods for Cox regression with right-truncated data. Biometrics 76(2):484–495 31621059 10.1111/biom.13162 PMC7162718

[CR37] Vakulenko-Lagun B, Qian J, Chiou SH, Wang N, Betensky RA (2022) Nonparametric estimation of the survival distribution under covariate-induced dependent truncation. Biometrics 78(4):1390–1401 34389985 10.1111/biom.13545

[CR38] Wang Y, Ying A, Xu R (2024) Doubly robust estimation under covariate-induced dependent left truncation. Biometrika p asae005 10.1093/biomet/asae005 PMC11648126 39691694

[CR39] Xiao J, Hudgens M (2019) On nonparametric maximum likelihood estimation with double truncation. Biometrika 106(4):989–996 31754284 10.1093/biomet/asz038 PMC6845852

[CR40] Zhou Y, Liu J, Zhu L (2020) Test for conditional independence with application to conditional screening. J Multivar Anal 175:104557

