# Supplementary material for: Robust inverse probability weighted estimators for doubly truncated Cox regression with closed-form standard errors
Source: Lifetime Data Anal. 2025 Apr 15;31(2):364–93. doi: 10.1007/s10985-025-09650-5 (PMC12043752; doi:10.1007/s10985-025-09650-5)
Supplement: Supplementary file 1 — (pdf 457 KB) [file 10985_2025_9650_MOESM1_ESM.pdf]

Supplementary Material: Robust inverse probability weighted estimators for doubly truncated Cox regression with closed-form standard errors

Abstract

Section S1 contains the influence function derivations for the NPMLE. In Section S2, we derive the asymptotic null distribution for the quasi-independent truncation diagnostic test statistic and describe how to sample from it. Then in Section S3 we include additional examples of nonparametric analyses that leverage our closed-form influence function estimates. In Section S4 we derive the influence functions for IPW Cox regression with time-varying weights, the sensitivity analysis estimator for possible violations of the positivity assumption, and the IPW baseline hazard estimator. Section S5 contains additional simulation results for the NPMLE, quasi-independent truncation diagnostic test, and robust IPW Cox regression estimators (including non-proportional hazards).

S1 NPMLE influence function estimator

Here we derive a simple expression for the plug-in estimator of the NPMLE influence function under the strong connectedness graphical condition described in the main text. Recall that the NPMLE $\widehat{F}(t) = \sum_{i=1}^n \widehat{\phi}_i \mathbf{1}(T_i \leq t)$ with point masses $\widehat{\boldsymbol{\phi}} = \{\widehat{\phi}_1, \ldots, \widehat{\phi}_n\}$ is defined as the minimizer of the negative log-likelihood $\ell_F(\boldsymbol{\phi}) = \sum_{i=1}^n [\log(\Phi_i) - \log(\phi_i)]$, with $\boldsymbol{\Phi} = \boldsymbol{J}^\intercal \boldsymbol{\phi}$, subject to the constraints $\phi_i > 0$, $i = 1, \ldots, n$, and $\sum_{i=1}^n \phi_i = 1$. Lemma S1 below characterizes the eigenvalues of the symmetric matrix $\left(\boldsymbol{S}\text{diag}(\widehat{\boldsymbol{\phi}})\right)^{-1} \boldsymbol{A} \left(\boldsymbol{S}\text{diag}(\widehat{\boldsymbol{\phi}})\right) = \text{diag}(\widehat{\boldsymbol{\phi}})\boldsymbol{J}\text{diag}(1/\widehat{\boldsymbol{\Phi}}^2)\boldsymbol{J}^\intercal\text{diag}(\widehat{\boldsymbol{\phi}})$. The influence function estimator is then derived in Theorem S1.

Lemma S1. *Under the strong connectedness condition, the largest eigenvalue of*

$$diag(\widehat{\boldsymbol{\phi}})\boldsymbol{J}\,diag(1/\widehat{\boldsymbol{\Phi}}^2)\boldsymbol{J}^\intercal\,diag(\widehat{\boldsymbol{\phi}})$$

is equal to one, with eigenvector $\mathbf{1}_n$*, and the remaining* $n-1$ *eigenvalues are contained in the interval* $[0, 1)$.

Proof. By the re-parameterization $\boldsymbol{z} = \log(\boldsymbol{\phi})$, the objective function to minimize becomes

$$\widetilde{\ell}_F(\boldsymbol{z}) = \sum_{i=1}^n [\log(\Phi_i) - \boldsymbol{z}_i]\,, \;\; \boldsymbol{\Phi} = \boldsymbol{J}^\intercal \exp(\boldsymbol{z}),$$

with score function $\nabla\widetilde{\ell}_F(\boldsymbol{z}) = \text{diag}(\exp(\boldsymbol{z}))\boldsymbol{J}\left(\frac{1}{\boldsymbol{\Phi}}\right) - \mathbf{1}_n$ and Hessian matrix

$$\nabla^2\widetilde{\ell}_F(\boldsymbol{z}) = \text{diag}(\exp(\boldsymbol{z}))\text{diag}(\boldsymbol{J}\,(1/\boldsymbol{\Phi})) - \text{diag}(\exp(\boldsymbol{z}))\boldsymbol{J}\text{diag}(1/\boldsymbol{\Phi}^2)\boldsymbol{J}^\top\text{diag}(\exp(\boldsymbol{z})).$$

For any n-vector $\boldsymbol{b}$, we have

$$\begin{aligned}\boldsymbol{b}^\top\nabla^2\widetilde{\ell}_F(\boldsymbol{z})\boldsymbol{b} &= \sum_{i=1}^n \boldsymbol{b}_i^2\exp(\boldsymbol{z}_i)\boldsymbol{J}_{i\cdot}\left(\frac{1}{\boldsymbol{\Phi}}\right) - \sum_{i=1}^n\sum_{j=1}^n \boldsymbol{b}_i\boldsymbol{b}_j\exp(\boldsymbol{z}_i)\boldsymbol{J}_{i\cdot}\text{diag}(1/\boldsymbol{\Phi}^2)\boldsymbol{J}_{j\cdot}^\top\exp(\boldsymbol{z}_j)\\ &= \sum_{k=1}^n\left[\sum_{i=1}^n \boldsymbol{b}_i^2\boldsymbol{p}_i(k) - \left(\sum_{i=1}^n \boldsymbol{b}_i\boldsymbol{p}_i(k)\right)^2\right]\end{aligned}$$

with $\boldsymbol{p}_i(k) = \exp(\boldsymbol{z}_i)\boldsymbol{J}_{ik}/\Phi_k \in [0,1)$. This sum is non-negative by the Cauchy-Schwarz Inequality, and is strictly positive unless $\boldsymbol{b}\sqrt{\boldsymbol{p}(k)}$ and $\sqrt{\boldsymbol{p}(k)}$ are linearly dependent for each k. Suppose this is true for some $\boldsymbol{b} \neq \mathbf{0}$, i.e. there exist constants $c_1,\dots,c_k$ such that $\boldsymbol{b}_i\sqrt{\boldsymbol{p}_i(k)} = c_k\sqrt{\boldsymbol{p}_i(k)}$ for all i and k. This implies that $\boldsymbol{b}_i = c_k = \boldsymbol{b}_k$ whenever $\boldsymbol{p}_i(k) > 0$, or equivalently when $\boldsymbol{J}_{ik} = 1$ (note $\boldsymbol{p}_k(k) > 0$ always). Therefore $\boldsymbol{b}_1 = \cdots = \boldsymbol{b}_n$ by the strong connectedness condition. In summary, $\nabla^2\widetilde{\ell}_F(\boldsymbol{z})$ is non-negative definite with rank $n-1$, and its null space is spanned by $\mathbf{1}_n$.

Returning to the original parameterization and evaluating at $\widehat{\boldsymbol{\phi}}$, we have

$$\begin{aligned}\nabla^2\widetilde{\ell}_F(\log(\widehat{\boldsymbol{\phi}})) &= \text{diag}(\widehat{\boldsymbol{\phi}})\text{diag}(\boldsymbol{J}(1/\widehat{\boldsymbol{\Phi}})) - \text{diag}(\widehat{\boldsymbol{\phi}})\boldsymbol{J}\text{diag}(1/\widehat{\boldsymbol{\Phi}}^2)\boldsymbol{J}^\top\text{diag}(\widehat{\boldsymbol{\phi}})\\ &= \boldsymbol{I}_n - \text{diag}(\widehat{\boldsymbol{\phi}})\boldsymbol{J}\text{diag}(1/\widehat{\boldsymbol{\Phi}}^2)\boldsymbol{J}^\top\text{diag}(\widehat{\boldsymbol{\phi}})\end{aligned}$$

where we use the fact that $\widehat{\boldsymbol{\phi}}$ is a root of the score function $\nabla\ell_F(\boldsymbol{\phi}) = \boldsymbol{J}\frac{1}{\boldsymbol{\Phi}} - \frac{1}{\boldsymbol{\phi}}$. Clearly the matrix $\text{diag}(\widehat{\boldsymbol{\phi}})\boldsymbol{J}\text{diag}(1/\widehat{\boldsymbol{\Phi}}^2)\boldsymbol{J}^\top\text{diag}(\widehat{\boldsymbol{\phi}})$ is non-negative definite, and, by the results for $\nabla^2\widetilde{\ell}_F(\boldsymbol{z})$ shown above, it has $n-1$ eigenvalues that are strictly less than one, while the remaining eigenvalue is equal to one (with associated eigenvector $\mathbf{1}_n$). □

Theorem S1 (Influence Function Estimator)**.** *Suppose the event times are sorted so that $T_1 \le T_2 \le \ldots \le T_n$. Under the strong connectedness condition, $\boldsymbol{I}_n - \boldsymbol{A}$ has all non-negative eigenvalues and rank $n-1$. The plug-in estimator of the influence function for $\widehat{F}$, $\boldsymbol{\mathcal{A}}_{ji} = \widehat{\mathcal{A}[h_i](T_j)}$, is contained in the rows of the unique solution of the matrix equation*

$$[\boldsymbol{I}_n - \boldsymbol{A}]\,\boldsymbol{X} = \boldsymbol{H}$$

subject to the constraint $\mathbf{1}_n^\top\left(\boldsymbol{S}\,\text{diag}(\widehat{\boldsymbol{\phi}})\right)^{-1}\boldsymbol{X} = \mathbf{0}_n^\top$.

Letting $\boldsymbol{B} = \boldsymbol{S}\,\text{diag}(\widehat{\boldsymbol{\phi}})$ and $\boldsymbol{C} = \begin{bmatrix}\boldsymbol{I}_{n-1}\\ -\mathbf{1}_{n-1}^\top\end{bmatrix}$, the estimator can be expressed as

$$\boldsymbol{\mathcal{A}} = \boldsymbol{I}^u\boldsymbol{B}\boldsymbol{C}\left[\boldsymbol{C}^\top\boldsymbol{B}^{-1}(\boldsymbol{I}_n - \boldsymbol{A})\,\boldsymbol{B}\boldsymbol{C}\right]^{-1}\boldsymbol{C}^\top\boldsymbol{B}^{-1}\boldsymbol{H}.$$

Proof. The exact plug-in estimator evaluated at time T_j is given by the kth row of $\sum_{r=0}^{\infty} \boldsymbol{A}^r \boldsymbol{H}$, where $T_k = T_j$ and either $k = n$ or $T_{k+1} \neq T_j$. By Lemma S1, the eigenvalues of

$$\boldsymbol{A} = \boldsymbol{S}\mathrm{diag}(\widehat{\boldsymbol{\phi}}) \left[\mathrm{diag}(\widehat{\boldsymbol{\phi}})\boldsymbol{J}\mathrm{diag}(1/\widehat{\boldsymbol{\Phi}}^2)\boldsymbol{J}^\mathsf{T}\mathrm{diag}(\widehat{\boldsymbol{\phi}})\right] \left(\boldsymbol{S}\mathrm{diag}(\widehat{\boldsymbol{\phi}})\right)^{-1},$$

lie within $[0, 1]$, and only one eigenvalue is strictly equal to one, with eigenvector $\boldsymbol{S}\mathrm{diag}(\widehat{\boldsymbol{\phi}})\mathbf{1}_n$. Thus the infinite sum is well-defined as long as $\mathbf{1}_n^\mathsf{T} \left(\boldsymbol{S}\mathrm{diag}(\widehat{\boldsymbol{\phi}})\right)^{-1} \boldsymbol{H} = \mathbf{0}_n^\mathsf{T}$. We can easily verify this condition since $\boldsymbol{H}_{ki} = n\widehat{\phi}_i \mathbf{1}(T_i \leq T_k) - n\sum_{s=1}^n \boldsymbol{J}_{si}\mathbf{1}(T_s \leq T_k)\widehat{\phi}_s^2/\widehat{\Phi}_i$ so

$$\mathbf{1}_n^\mathsf{T} \left(\boldsymbol{S}\mathrm{diag}(\widehat{\boldsymbol{\phi}})\right)^{-1} \boldsymbol{H}_{\cdot i}/n = \frac{\boldsymbol{H}_{1i}}{n\widehat{\phi}_1} + \sum_{k=2}^{n} \frac{\boldsymbol{H}_{ki} - \boldsymbol{H}_{k-1,i}}{n\widehat{\phi}_k}$$

$$= \sum_{k=1}^{n} \left[\frac{\widehat{\phi}_i \mathbf{1}(T_i \in (T_{k-1}, T_k])}{\widehat{\phi}_k} - \frac{\sum_{s=1}^n \boldsymbol{J}_{si}\mathbf{1}(T_s \in (T_{k-1}, T_k])\widehat{\phi}_s^2}{\widehat{\Phi}_i\widehat{\phi}_k}\right]$$

$$= 1 - \sum_{s=1}^{n} \frac{\boldsymbol{J}_{si}\widehat{\phi}_s^2}{\widehat{\Phi}_i} \sum_{k=1}^{n} \frac{\mathbf{1}(T_s \in (T_{k-1}, T_k])}{\widehat{\phi}_k} = 1 - \sum_{s=1}^{n} \frac{\boldsymbol{J}_{si}\widehat{\phi}_s}{\widehat{\Phi}_i} = 0$$

where we define $T_0 = -\infty$ and $\mathbf{1}(T_i \in (T_{k-1}, T_k]) = 0$ if $T_{k-1} = T_k$.

Let $\boldsymbol{P}\boldsymbol{\Lambda}\boldsymbol{P}^\mathsf{T}$, where $\boldsymbol{P}$ is orthogonal and $\boldsymbol{\Lambda}$ is a diagonal matrix, be the eigendecomposition of the symmetric non-negative definite matrix $\mathrm{diag}(\widehat{\boldsymbol{\phi}})\boldsymbol{J}\mathrm{diag}(1/\widehat{\boldsymbol{\Phi}}^2)\boldsymbol{J}^\mathsf{T}\mathrm{diag}(\widehat{\boldsymbol{\phi}})$. We have

$$\sum_{r=0}^{\infty} \boldsymbol{A}^r \boldsymbol{H} = \boldsymbol{S}\mathrm{diag}(\widehat{\boldsymbol{\phi}})\boldsymbol{P}\left[\boldsymbol{I}_n - \boldsymbol{\Lambda}\right]^- \boldsymbol{P}^\mathsf{T} \left(\boldsymbol{S}\mathrm{diag}(\widehat{\boldsymbol{\phi}})\right)^{-1} \boldsymbol{H}$$

where $[\boldsymbol{I}_n - \boldsymbol{\Lambda}]^-$ is a diagonal matrix with entries $(1 - \boldsymbol{\Lambda}_{ii})^{-1}$ for $\boldsymbol{\Lambda}_{ii} > 0$, and 0 for $\boldsymbol{\Lambda}_{ii} = 0$. Note that this is the unique solution of the matrix equation $(\boldsymbol{I}_n - \boldsymbol{A})\boldsymbol{X} = \boldsymbol{H}$ under the constraint that each column of $\left(\boldsymbol{S}\mathrm{diag}(\widehat{\boldsymbol{\phi}})\right)^{-1} \boldsymbol{X}$ is orthogonal to the null space of $\boldsymbol{P}\left[\boldsymbol{I}_n - \boldsymbol{\Lambda}\right]\boldsymbol{P}^\mathsf{T}$, which is spanned by $\mathbf{1}_n$.

Lastly, we can avoid directly solving a rank-deficient matrix equation by using the fact that any n-vector $\boldsymbol{x}$ such that $\mathbf{1}_n^\mathsf{T}\boldsymbol{x} = 0$ can be represented by its first $n-1$ entries. For $\boldsymbol{C} = \begin{bmatrix} \boldsymbol{I}_{n-1} \\ -\mathbf{1}_{n-1}^\mathsf{T} \end{bmatrix}$ we have $\boldsymbol{x} = (\boldsymbol{x}_1, \ldots \boldsymbol{x}_{n-1},\ -\sum_{k=1}^{n-1} \boldsymbol{x}_k)^\mathsf{T} = \boldsymbol{C}\boldsymbol{x}_{-n}$. Note that $\boldsymbol{C}$ has rank $n-1$ and $\boldsymbol{C}^\mathsf{T}\mathbf{1}_n = \mathbf{0}_n$. Then $(\boldsymbol{I}_n - \boldsymbol{A})\boldsymbol{X} = \boldsymbol{H}$ with $\mathbf{1}_n^\mathsf{T} \left(\boldsymbol{S}\mathrm{diag}(\widehat{\boldsymbol{\phi}})\right)^{-1} \boldsymbol{X} = \mathbf{0}_n^\mathsf{T}$ if and only if $\boldsymbol{P}(\boldsymbol{I}_n - \boldsymbol{\Lambda})\boldsymbol{P}^\mathsf{T}\boldsymbol{C}\boldsymbol{Y}_{-n,\cdot} = (\boldsymbol{S}\mathrm{diag}(\widehat{\boldsymbol{\phi}}))^{-1}\boldsymbol{H}$, where $\boldsymbol{Y} = (\boldsymbol{S}\mathrm{diag}(\widehat{\boldsymbol{\phi}}))^{-1}\boldsymbol{X}$. The columns of each side are orthogonal to $\mathbf{1}_n$ and $\boldsymbol{P}(\boldsymbol{I}_n - \boldsymbol{\Lambda})\boldsymbol{P}^\mathsf{T}\boldsymbol{C}$ has full rank, so the solution is

$$\boldsymbol{X} = \boldsymbol{S}\mathrm{diag}(\widehat{\boldsymbol{\phi}})\boldsymbol{Y} = \boldsymbol{S}\mathrm{diag}(\widehat{\boldsymbol{\phi}})\boldsymbol{C}\boldsymbol{Y}_{-n,\cdot}$$

$$= \boldsymbol{S}\mathrm{diag}(\widehat{\boldsymbol{\phi}})\boldsymbol{C}\left(\boldsymbol{C}^\mathsf{T}\boldsymbol{P}(\boldsymbol{I}_n - \boldsymbol{\Lambda})\boldsymbol{P}^\mathsf{T}\boldsymbol{C}\right)^{-1}\boldsymbol{C}^\mathsf{T}(\boldsymbol{S}\mathrm{diag}(\widehat{\boldsymbol{\phi}}))^{-1}\boldsymbol{H}.$$

□

S2 Quasi-independent truncation diagnostic asymptotics

Here we derive the asymptotic null distribution of the (scaled) nonparametric quasi-independent truncation test statistic $\sqrt{n}\sup_{s,t}|\widehat{\pi}(t|s)-\widehat{\pi}(t)|$, where $\widehat{\pi}(t|s)$ is the sth stratum-specific NPMLE for the selection probabilities and $\widehat{\pi}(t)$ is the unstratified NPMLE. Under quasi-independent truncation, $\sup_{s,t}|\widehat{\pi}(t|s)-\widehat{\pi}(t)|$ should tend to zero since the selection probabilities $\pi(t)=\int \mathbf{1}(t\in[u,v])dK(u,v)$ would not depend on the covariate stratum. We show how to sample from the asymptotic null distribution in order to construct a p-value for the diagnostic test as well as a confidence band for $\widehat{\pi}(t|s)-\widehat{\pi}(t)$ in the graphical diagnostic.

First, we derive the asymptotic distribution of the NPMLE $\widehat{\pi}(t)$ in Lemma S2 below. Then, similar to Section S1, we provide a simple closed-form expression for the empirical influence function of $\widehat{\pi}(t)$ in Lemma S3. We conclude by describing how to sample from the asymptotic null distribution of $\sqrt{n}\sup_{s,t}|\widehat{\pi}(t|s)-\widehat{\pi}(t)|$.

Lemma S2 (Asymptotic Distribution of $\widehat{\pi}(t)$)**.** *Under assumptions (1)-(3) in the main text, the NPMLE of the selection probabilities $\widehat{\pi}(t)$ satisfies*

$$\sqrt{n}\left(\widehat{\pi}(t)-\pi(t)\right)=\frac{1}{\sqrt{n}}\sum_{i=1}^{n}\left[L_i^a(t)\alpha+a(t)L_i^\alpha\right]+o_p(1)$$

where

$$L_i^\alpha=-\alpha^2\left\{\frac{1}{F(V_i)-F(U_i-)}-\frac{1}{\alpha}-\int\frac{\mathcal{A}[h_i](v)-\mathcal{A}[h_i](u-)}{[F(v)-F(u-)]^2}dK^*(u,v)\right\}.$$

and

$$L_i^a(t)=\frac{\mathbf{1}(t\in[U_i,V_i])}{F(V_i)-F(U_i-)}-a(t)-\widetilde{\mathcal{A}}[h_i](t).$$

Thus $\sqrt{n}\left(\widehat{\pi}(t)-\pi(t)\right)$ converges weakly to a mean-zero Gaussian process $G(t)$ with

$$\mathbb{E}^*[G(t)G(s)]=\mathbb{E}^*\left[\left(L_i^a(t)\alpha+a(t)L_i^\alpha\right)\left(L_i^a(s)\alpha+a(s)L_i^\alpha\right)\right].$$

Proof. First, recall the overall probability of non-truncation is $\alpha=\mathbb{P}(U\le T\le V)=\{\int[F(v)-F(u-)]^{-1}dK^*(u,v)\}^{-1}$, with estimate $\widehat{\alpha}=\{\int[\widehat{F}(v)-\widehat{F}(u-)]^{-1}d\widehat{K^*}(u,v)\}^{-1}$, where $\widehat{K^*}(u,v)$ is the empirical CDF for the bivariate truncation time distribution. Then, recalling that $\widehat{F}(t)-F(t)=n^{-1}\sum_{i=1}^{n}\mathcal{A}[h_i](t)+o_p\left(1/\sqrt{n}\right)$, by Slutsky's Theorem we have

$$\begin{aligned}\frac{1}{\widehat{\alpha}}-\frac{1}{\alpha}&=\int\frac{d(\widehat{K^*}-K^*)(u,v)}{\widehat{F}(v)-\widehat{F}(u-)}-\int\frac{[\widehat{F}(v)-F(v)-\widehat{F}(u-)+F(u-)]dK^*(u,v)}{[\widehat{F}(v)-\widehat{F}(u-)][F(v)-F(u-)]}\\&=\int\frac{d(\widehat{K^*}-K^*)(u,v)}{F(v)-F(u-)}-\int\frac{[\widehat{F}(v)-F(v)-\widehat{F}(u-)+F(u-)]dK^*(u,v)}{[F(v)-F(u-)]^2}+o_p(1/\sqrt{n})\\&=\frac{1}{n}\sum_{i=1}^{n}\left\{\frac{1}{F(V_i)-F(U_i-)}-\frac{1}{\alpha}-\int\frac{\mathcal{A}[h_i](v)-\mathcal{A}[h_i](u-)}{[F(v)-F(u-)]^2}dK^*(u,v)\right\}+o_p(1/\sqrt{n}).\end{aligned}$$

Therefore, by the Delta method, $\widehat{\alpha} - \alpha = n^{-1} \sum_{i=1}^{n} L_i^{\alpha} + o_p(1/\sqrt{n})$ where

$$L_i^{\alpha} = -\alpha^2 \left\{ \frac{1}{F(V_i) - F(U_i-)} - \frac{1}{\alpha} - \int \frac{\mathcal{A}[h_i](v) - \mathcal{A}[h_i](u-)}{[F(v) - F(u-)]^2} dK^*(u,v) \right\}.$$

Additionally, de Uña-Álvarez and Keilegom (2021) showed that $\widehat{a}(t) - a(t) = n^{-1} \sum_{i=1}^{n} L_i^{a}(t) + o_p(1/\sqrt{n})$ with

$$L_i^{a}(t) = \frac{\mathbf{1}(t \in [U_i, V_i])}{F(V_i) - F(U_i-)} - a(t) - \widetilde{\mathcal{A}}[h_i](t).$$

Then, the NPMLE of the selection probabilities $\widehat{\pi}(t) = \widehat{a}(t)\widehat{\alpha}$ satisfies

$$\begin{aligned} \widehat{\pi}(t) - \pi(t) &= [\widehat{a}(t) - a(t)]\widehat{\alpha} + a(t)[\widehat{\alpha} - \alpha] \\ &= \frac{1}{n} \sum_{i=1}^{n} [L_i^{a}(t)\alpha + a(t)L_i^{\alpha}] + o_p(1/\sqrt{n}) \end{aligned}$$

where $\{L_i^{a}(t)\alpha + a(t)L_i^{\alpha}\}$ is a Donsker class. This completes the proof. □

Lemma S3 (Influence Function Estimator)**.** *Under the strong connectedness condition, the plug-in estimates for the influence function $L_i^{a}(t)\alpha + a(t)L_i^{\alpha}$ at time t are given by the ith column of*

$$[\widehat{\alpha}\boldsymbol{J}_t - \widehat{a}(t)\widehat{\alpha}^2\mathbf{1}_n]^{\intercal}[\mathit{diag}(1/\widehat{\boldsymbol{\Phi}}) - \mathit{diag}(1/\widehat{\boldsymbol{\Phi}}^2)\boldsymbol{J}^{\intercal}\boldsymbol{S}^{-1}\boldsymbol{\mathcal{A}}/n]$$

where $\boldsymbol{J}_t$ is an n-vector with ith entry $\mathbf{1}(t \in [U_i, V_i])$ and both $\boldsymbol{S}$ and $\boldsymbol{\mathcal{A}}$ are defined in Lemma 1 of the main text.

Proof. By directly plugging-in all NPMLE estimates in the expressions for $L_i^{a}(t)$ and L_i^{α} in Lemma S2, the estimate for $L_i^{a}(t)\alpha + a(t)L_i^{\alpha}$ becomes the ith column of

$$\begin{aligned} &[\boldsymbol{J}_t^{\intercal}\mathrm{diag}(1/\widehat{\boldsymbol{\Phi}}) - \widehat{a}(t)\mathbf{1}_n^{\intercal} - \boldsymbol{J}_t^{\intercal}\mathrm{diag}(1/\widehat{\boldsymbol{\Phi}}^2)\boldsymbol{J}^{\intercal}\boldsymbol{S}^{-1}\boldsymbol{\mathcal{A}}/n]\widehat{\alpha} \\ &\quad - \widehat{a}(t)\widehat{\alpha}^2[\mathbf{1}_n^{\intercal}\mathrm{diag}(1/\widehat{\boldsymbol{\Phi}}) - \widehat{\alpha}^{-1}\mathbf{1}_n^{\intercal} - \mathbf{1}_n^{\intercal}\mathrm{diag}(1/\widehat{\boldsymbol{\Phi}}^2)\boldsymbol{J}^{\intercal}\boldsymbol{S}^{-1}\boldsymbol{\mathcal{A}}/n] \\ &= [\widehat{\alpha}\boldsymbol{J}_t - \widehat{a}(t)\widehat{\alpha}^2\mathbf{1}_n]^{\intercal}[\mathrm{diag}(1/\widehat{\boldsymbol{\Phi}}) - \mathrm{diag}(1/\widehat{\boldsymbol{\Phi}}^2)\boldsymbol{J}^{\intercal}\boldsymbol{S}^{-1}\boldsymbol{\mathcal{A}}/n]. \end{aligned}$$

□

We now describe how to sample from the asymptotic null distribution of the test statistic $\sqrt{n}\sup_{s,t}|\widehat{\pi}(t|s) - \widehat{\pi}(t)|$. The null hypothesis here is that quasi-independent truncation holds, so $\pi(t|s) = \pi(t)$ for all times t and each covariate stratum s. Let s_i be the stratum id for the ith data point. By Lemma S2 above, $\widehat{\pi}(t) - \pi(t) = n^{-1}\sum_{i=1}^{n} L_i^{\pi}(t) + o_p(1/\sqrt{n})$ for some function $L_i^{\pi}(t)$ with an estimate provided by Lemma S3. Similarly, $\widehat{\pi}(t|s) - \pi(t) = n_s^{-1}\sum_{s_i=s} L_i^{\pi}(t|s) + o_p(1/\sqrt{n})$ where $n_s = \sum_{i=1}^{n}\mathbf{1}(s_i = s)$ is the stratum size and $L_i^{\pi}(t|s)$ can be estimated as in Lemma S3 using data from only the sth stratum. Then

$$\sqrt{n}\,(\widehat{\pi}(t|s) - \widehat{\pi}(t)) = n^{-1/2}\sum_{i=1}^{n}[(n/n_s)\mathbf{1}(s_i = s)L_i^{\pi}(t|s) - L_i^{\pi}(t)] + o_p(1)$$

which converges weakly to a mean zero Gaussian process. To sample from this process, let the matrix $\boldsymbol{L}^{\pi}$ contain the estimates for $(n/n_s)\mathbf{1}(s_i = s)L_i^{\pi}(t|s) - L_i^{\pi}(t)$, with individuals $i = 1, \ldots, n$ contained in the n columns and every combination of stratum $s \in \{1, \ldots, S\}$ and time $t \in [a_T, b_T]$ in the rows. Note that it suffices to only consider t at the observed truncation times (within the event time support $[a_T, b_T]$), since the influence function will be constant between observed truncation times. Generate a weight vector $\boldsymbol{w}$ with iid entries that have variance one, independent of the observed data, e.g. $N(0,1)$ or Exponential(1) for heaver tails (the mean may be arbitrary since the influence function estimates sum to zero over individuals, and the weights do not need to be non-negative). Then $\max|\boldsymbol{L}^{\pi}\boldsymbol{w}/n|$ is a sample from the estimated null distribution of $\sup_{s,t}|\widehat{\pi}(t|s) - \widehat{\pi}(t)|$ since, conditional on the data, $\boldsymbol{L}^{\pi}\boldsymbol{w}/n$ is approximately normal (by the CLT) with mean $\propto \boldsymbol{L}^{\pi}\mathbf{1}_n/n = \mathbf{0}$ and covariance $\boldsymbol{L}^{\pi}(\boldsymbol{L}^{\pi})^{\mathsf{T}}/n$.

S3 Additional nonparametric analyses

Example S1 (Nonparametric cumulative hazard estimation)**.** *Let $\widehat{S}(t) = 1 - \widehat{F}(t)$ be the nonparametric estimator of the survival function $S(t) = 1 - F(t)$. In survival analysis, one is often interested in the cumulative hazard rate $\Lambda(t) = \int_{a_T}^t S(t-)^{-1}dF(t)$, rather than in the survival function itself. Using the NPMLE $\widehat{F}(t)$, the cumulative hazard estimate is $\widehat{\Lambda}(t) = \int_{a_T}^t \widehat{S}(u-)^{-1}d\widehat{F}(u)$. By the functional delta method we have*

$$\widehat{\Lambda}(t) - \Lambda(t) = \frac{1}{n}\sum_{i=1}^{n}\left[\int_{a_T}^{t}\frac{d\mathcal{A}[h_i](u)}{S(u-)} + \int_{a_T}^{t}\frac{\mathcal{A}[h_i](u-)}{S(u-)^2}dF(u)\right] + o_p\left(1/\sqrt{n}\right)$$

uniformly in $t \in [a_T, \tau]$, where $\tau < b_T$ is chosen such that $S(\tau-) > 0$. The plug-in estimate for this influence function follows easily from the results of Lemma 1 in the main text, which can then be used to construct pointwise confidence intervals for $\widehat{\Lambda}(t)$ based on a normal approximation or uniform confidence bands by sampling from the estimated asymptotic distribution.

Example S2 (Two-sample tests)**.** *Suppose we are interested in comparing the hazard rates of two independent samples, with size n_1 and n_2 respectively. Let $\widehat{\Lambda}_1(t)$ and $\widehat{\Lambda}_2(t)$ be the nonparametric cumulative hazard estimates for the two samples, as described above, and $w(t)$ be a positive function of bounded variation. Suppose an asymptotically linear estimator $\widehat{w}(t)$ is available. In the spirit of Fleming et al. (1987), consider the integrated weighted difference*

$$D^w(t) = \int_{a_T}^{t}\widehat{w}(u)d(\widehat{\Lambda}_1 - \widehat{\Lambda}_2)(u)$$

for $t \in [a_T, \tau]$ such that $\min(S_1(\tau-), S_2(\tau-)) > 0$.

Let $n_1/(n_1+n_2) \to \lambda \in [0,1]$. Under $H_0 : \Lambda_1 = \Lambda_2$, $\sqrt{\lambda n_2}D^w(t)$ converges weakly to a weighted sum of independent mean-zero Gaussian processes $\sqrt{1-\lambda}G_1(t) + \sqrt{\lambda}G_2(t)$ as $n_1, n_2 \to \infty$. Here $Cov(G_j(t), G_j(s)) = \mathbb{E}^*[\int_{a_T}^t w(u)dL_{ij}(u)\int_{a_T}^s w(u)dL_{ij}(u)]$, where $L_{ij}(t)$ is the influence function for $\widehat{\Lambda}_j(t)$. This determines the null distribution of the test statistic $\sup_{t\in[a_T,\tau]}|\sqrt{\lambda n_2}D^w(t)|$, for which p-values can then be computed by simulation.

S4 IPW Cox regression asymptotics

In this section, we derive the influence function for the IPW Cox regression estimator $\widehat{\boldsymbol{\beta}}_w$ with time-varying weight function $w(\cdot)$ (Theorem S2), as well as for the corresponding sensitivity analysis estimator $\widehat{\boldsymbol{\beta}}_{w,p_{r|l}}$ with assumed truncated mass $p_{r|l}$ (Theorem S3). We assume that $\widehat{\boldsymbol{\beta}}_w \to_p \boldsymbol{\beta}^*_w$ and $\widehat{\boldsymbol{\beta}}_{w,p_{r|l}} \to_p \boldsymbol{\beta}^*_{w,p_{r|l}}$ for some well-defined constants $\boldsymbol{\beta}^*_w$ and $\boldsymbol{\beta}^*_{w,p_{r|l}}$. This allows for misspecification of the fitted model as well as of the truncated mass $p_{r|l}$. Lastly, we derive the influence function for the IPW baseline hazard estimator in Theorem S4.

Theorem S2 (IPW Cox Regression Asymptotics for $\widehat{\boldsymbol{\beta}}_w$)**.** *Define*

$$\Lambda_i(t) = \int_{a_T}^{t} Y_i(s)\exp(\boldsymbol{x}_i(s)^\mathsf{T}\boldsymbol{\beta}^*_w)d\Lambda^*_w(s),\ \ M_i(t) = N_i(t) - \Lambda_i(t)$$

where $\Lambda^*_w(t) = \mathbb{E}^*\left[\int_{a_T}^{t} \frac{dN(s)}{a(T)\boldsymbol{s}^{(0)}_{\boldsymbol{a}}(s,\boldsymbol{\beta}^*_w)}\right]$. *Let* $L_i(t)$ *be the influence function for* $1/\widehat{a}(t)$ *at data point* $\boldsymbol{D}_i$.

Then under assumptions (1)-(7) in the main text, replacing $\boldsymbol{\beta}^0$ *and* Λ_0 *with* $\boldsymbol{\beta}^*_w$ *and* Λ^*_w,

$$\widehat{\boldsymbol{\beta}}_w - \boldsymbol{\beta}^*_w = \frac{1}{n}\sum_{i=1}^{n} \boldsymbol{\mathcal{I}}_w^{-1}\left(\boldsymbol{U}_{w1i} + \boldsymbol{U}_{w2i} + \boldsymbol{U}_{w3i}\right) + o_p\left(1/\sqrt{n}\right)$$

where

$$\boldsymbol{U}_{w1i} = \int_{a_T}^{b_T} \frac{w(t)}{a(T_i)}\left[\boldsymbol{x}_i(t) - \boldsymbol{e}_{\boldsymbol{a}}(t,\boldsymbol{\beta}^*_w)\right] dM_i(t),$$

$$\boldsymbol{U}_{w2i} = \mathbb{E}^*[L_i(T_0)a(T_0)\boldsymbol{U}_{w10}|\boldsymbol{D}_i]$$

for $\boldsymbol{D}_0$ *independent of* $\boldsymbol{D}_i$, $i = 1, \ldots, n$, *and*

$$\boldsymbol{U}_{w3i} = \mathbb{E}^*\left[\int_{a_T}^{b_T} \frac{L^w_i(t)}{a(T_0)}\left[\boldsymbol{x}_0(t) - \boldsymbol{e}_{\boldsymbol{a}}(t,\boldsymbol{\beta}^*_w)\right] dN_0(t)\Big|\boldsymbol{D}_i\right].$$

Proof. The proof for Theorem S2 is given in Appendix A of the main text. □

For the sensitivity analysis estimator $\widehat{\boldsymbol{\beta}}_{w,p_{r|l}}$ under possible violation of the positivity assumption, suppose the event time distribution is truncated to the interval $[L,R]$. We denote the law of $(T,U,V,\boldsymbol{x})|\{T \in [L,R]\}$ by $\widetilde{\mathbb{P}}$, while $\mathbb{P}^*$ is still the law of the observed data. Then we define $\widetilde{w}(t)$ to be the limit of $\widehat{w}(t)$, $\widetilde{a}(t) = \widetilde{\mathbb{P}}(t \in [U,V])/\widetilde{\mathbb{P}}(T \in [U,V])$, $\widetilde{\boldsymbol{s}}^{(r)}_{\boldsymbol{a}}(t,\boldsymbol{\beta}) = \widetilde{\mathbb{E}}[\boldsymbol{x}_i^{\otimes r}\exp(\boldsymbol{x}_i^\mathsf{T}\boldsymbol{\beta})\widetilde{Y}(t;\boldsymbol{\beta},p_{r|l})]$ for $r = 0,1,2$, and $\widetilde{\boldsymbol{e}}_{\boldsymbol{a}}(t,\boldsymbol{\beta}) = \widetilde{\boldsymbol{s}}^{(1)}_{\boldsymbol{a}}(t,\boldsymbol{\beta})/\widetilde{\boldsymbol{s}}^{(0)}_{\boldsymbol{a}}(t,\boldsymbol{\beta})$. We sometimes suppress the dependence on $p_{r|l}$ to simplify the notation. Lastly, let $\boldsymbol{\mathcal{I}}_{w,p_{r|l}}$ be the limit of $-\boldsymbol{Dr}(\boldsymbol{\beta}^*_{w,p_{r|l}};\widehat{\boldsymbol{a}},\widehat{w})$. We assume $\boldsymbol{\mathcal{I}}_{w,p_{r|l}}$ has full rank.

Theorem S3 (IPW Cox Regression Asymptotics for $\widehat{\boldsymbol{\beta}}_{w,p_{r|l}}$)**.** *Define*

$$\Lambda_i(t) = \int_{a_T}^{t} \widetilde{Y}_i(s;\boldsymbol{\beta}^*_{w,p_{r|l}},p_{r|l})\exp(\boldsymbol{x}_i^\mathsf{T}\boldsymbol{\beta}^*_w)d\Lambda^*_{w,p_{r|l}}(s),\ \ M_i(t) = N_i(t) - \Lambda_i(t)$$

where $\Lambda^*_{w,p_{r|l}}(t) = \mathbb{E}^*\left[\int_{a_T}^t \frac{dN(s)}{\widetilde{a}(T)\widetilde{\boldsymbol{s}}^{(0)}_{\boldsymbol{a}}(s,\boldsymbol{\beta}^*_{w,p_{r|l}})}\right]$. *Let* $L_i(t)$ *be the influence function for* $1/\widehat{a}(t)$ *at data point* $\boldsymbol{D}_i$.

Then, under assumptions analogous to those in Theorem S2,

$$\widehat{\boldsymbol{\beta}}_{w,p_{r|l}} - \boldsymbol{\beta}^*_{w,p_{r|l}} = \frac{1}{n}\sum_{i=1}^n \boldsymbol{\mathcal{I}}^{-1}_{w,p_{r|l}}\left(\boldsymbol{U}_{w1i} + \boldsymbol{U}_{w2i} + \boldsymbol{U}_{w3i}\right) + o_p\left(1/\sqrt{n}\right)$$

where

$$\boldsymbol{U}_{w1i} = \int_{a_T}^{b_T} \frac{\widetilde{w}(t)}{\widetilde{a}(T_i)}\left[\boldsymbol{x}_i - \widetilde{\boldsymbol{e}}_{\boldsymbol{a}}(t,\boldsymbol{\beta}^*_{w,p_{r|l}})\right] dM_i(t),$$

$$\boldsymbol{U}_{w2i} = \mathbb{E}^*[L_i(T_0)\widetilde{a}(T_0)\boldsymbol{U}_{w10}|\boldsymbol{D}_i]$$

for $\boldsymbol{D}_0$ *independent of* $\boldsymbol{D}_i$, $i = 1,\ldots,n$, *and*

$$\boldsymbol{U}_{w3i} = \mathbb{E}^*\left[\int_{a_T}^{b_T} \frac{L_i^w(t)}{\widetilde{a}(T_0)}\left[\boldsymbol{x}_0 - \widetilde{\boldsymbol{e}}_{\boldsymbol{a}}(t,\boldsymbol{\beta}^*_{w,p_{r|l}})\right] dN_0(t)\middle|\boldsymbol{D}_i\right].$$

Proof. The proof of Theorem S3 follows by the same arguments as Theorem S2. In particular, the process $n^{-1/2}\sum_{i=1}^n \widetilde{a}(T_i)^{-1}M_i(t)$ is again constructed to have uniform mean zero.

□

Finally, we derive the influence function of the IPW baseline hazard estimator, focusing on the case of correct model specification.

Theorem S4 (Asymptotics for Baseline Hazard Estimator)**.** *Define*

$$\Lambda_i(t) = \int_{a_T}^t Y_i(s)\exp(\boldsymbol{x}_i(s)^\mathsf{T}\boldsymbol{\beta}^0)d\Lambda_0(s),\ \ M_i(t) = N_i(t) - \Lambda_i(t).$$

Suppose $\widehat{\boldsymbol{\beta}} - \boldsymbol{\beta}^0 = \frac{1}{n}\sum_{i=1}^n L_i^{\boldsymbol{\beta}} + o_p(1/\sqrt{n})$. *Let* $L_i(t)$ *be the influence function for* $1/\widehat{a}(t)$ *at data point* $\boldsymbol{D}_i$.

Then, under assumptions (1)-(7) in the main text, the IPW baseline hazard estimator

$$\widehat{\Lambda}_0(t;\widehat{\boldsymbol{\beta}},\widehat{\boldsymbol{a}}) = \frac{1}{n}\sum_{i=1}^n \int_{a_T}^t \frac{\widehat{a}(T_i)^{-1}dN_i(s)}{\boldsymbol{S}^{(0)}_{\widehat{\boldsymbol{a}}}(s,\widehat{\boldsymbol{\beta}})}$$

satisfies

$$\widehat{\Lambda}_0(t;\widehat{\boldsymbol{\beta}},\widehat{\boldsymbol{a}}) - \Lambda_0(t) = \frac{1}{n}\sum_{i=1}^n\left[L_i^{\Lambda,1}(t) + L_i^{\Lambda,2}(t) + L_i^{\Lambda,3}(t)\right] + o_p\left(1/\sqrt{n}\right)$$

uniformly in $t \in [a_T,\tau]$, *where*

$$L_i^{\Lambda,1}(t) = -\left[\int_{a_T}^t \boldsymbol{e}_{\boldsymbol{a}}(s,\boldsymbol{\beta}^0)d\Lambda_0(s)\right]^\mathsf{T} L_i^{\boldsymbol{\beta}},$$

$$L_i^{\Lambda,2}(t) = \mathbb{E}^*\left[L_i(T_0)\int_{a_T}^t \frac{dM_0(s)}{\boldsymbol{s}_{\boldsymbol{a}}^{(0)}(s,\boldsymbol{\beta}^0)}\Big|\boldsymbol{D}_i\right]$$

for an independent observation $\boldsymbol{D}_0$ from the post-truncation law, and

$$L_i^{\Lambda,3}(t) = \int_{a_T}^t \frac{a(T_i)^{-1}dM_i(s)}{\boldsymbol{s}_{\boldsymbol{a}}^{(0)}(s,\boldsymbol{\beta}^0)}.$$

Proof. We decompose the baseline hazard estimator, which is an average of uniformly bounded entries, as

$$\widehat{\Lambda}_0(t;\widehat{\boldsymbol{\beta}},\widehat{\boldsymbol{a}}) = \frac{1}{n}\sum_{i=1}^n \int_{a_T}^t \frac{\widehat{a}(T_i)^{-1}dN_i(s)}{\boldsymbol{S}_{\widehat{\boldsymbol{a}}}^{(0)}(s,\widehat{\boldsymbol{\beta}})}$$

$$= \underbrace{\frac{1}{n}\sum_{i=1}^n \int_{a_T}^t \left[\frac{1}{\boldsymbol{S}_{\widehat{\boldsymbol{a}}}^{(0)}(s,\widehat{\boldsymbol{\beta}})} - \frac{1}{\boldsymbol{S}_{\widehat{\boldsymbol{a}}}^{(0)}(s,\boldsymbol{\beta}^0)}\right]\widehat{a}(T_i)^{-1}dN_i(s)}_{S_1} + \underbrace{\frac{1}{n}\sum_{i=1}^n \int_{a_T}^t \frac{\widehat{a}(T_i)^{-1}d\Lambda_i(s)}{\boldsymbol{S}_{\widehat{\boldsymbol{a}}}^{(0)}(s,\boldsymbol{\beta}^0)}}_{S_2}$$

$$+ \underbrace{\frac{1}{n}\sum_{i=1}^n \int_{a_T}^t \left[\frac{\widehat{a}(T_i)^{-1}}{\boldsymbol{S}_{\widehat{\boldsymbol{a}}}^{(0)}(s,\boldsymbol{\beta}^0)} - \frac{a(T_i)^{-1}}{\boldsymbol{S}_{\boldsymbol{a}}^{(0)}(s,\boldsymbol{\beta}^0)}\right]dM_i(s)}_{S_3} + \underbrace{\frac{1}{n}\sum_{i=1}^n \int_{a_T}^t \frac{a(T_i)^{-1}dM_i(s)}{\boldsymbol{S}_{\boldsymbol{a}}^{(0)}(s,\boldsymbol{\beta}^0)}}_{S_4}.$$

For S_1, by the convergence of $\widehat{\boldsymbol{\beta}}$ and the delta method we have

$$\boldsymbol{S}_{\widehat{\boldsymbol{a}}}^{(0)}(s,\widehat{\boldsymbol{\beta}}) - \boldsymbol{S}_{\widehat{\boldsymbol{a}}}^{(0)}(s,\boldsymbol{\beta}^0) = \frac{1}{n}\sum_{j=1}^n \widehat{a}(T_j)^{-1}Y_j(s)\left[\exp\left(\boldsymbol{x}_j(s)^\intercal\widehat{\boldsymbol{\beta}}\right) - \exp\left(\boldsymbol{x}_j(s)^\intercal\boldsymbol{\beta}^0\right)\right]$$

$$= \frac{1}{n}\sum_{j=1}^n \widehat{a}(T_j)^{-1}Y_j(s)\exp\left(\boldsymbol{x}_j(s)^\intercal\boldsymbol{\beta}^0\right)\boldsymbol{x}_j(s)^\intercal(\widehat{\boldsymbol{\beta}}-\boldsymbol{\beta}^0) + o_p\left(1/\sqrt{n}\right)$$

so

$$S_1 = -\frac{1}{n}\sum_{i=1}^n \int_{a_T}^t \frac{\boldsymbol{S}_{\widehat{\boldsymbol{a}}}^{(1)}(s,\boldsymbol{\beta}^0)^\intercal(\widehat{\boldsymbol{\beta}}-\boldsymbol{\beta}^0)\widehat{a}(T_i)^{-1}dN_i(s)}{\boldsymbol{S}_{\widehat{\boldsymbol{a}}}^{(0)}(s,\widehat{\boldsymbol{\beta}})\boldsymbol{S}_{\widehat{\boldsymbol{a}}}^{(0)}(s,\boldsymbol{\beta}^0)} + o_p\left(1/\sqrt{n}\right)$$

$$= -\frac{1}{n}\sum_{i=1}^n \int_{a_T}^t \frac{\overline{\boldsymbol{x}}_{\boldsymbol{a}}(s,\boldsymbol{\beta}^0)^\intercal a(T_i)^{-1}dN_i(s)}{\boldsymbol{S}_{\boldsymbol{a}}^{(0)}(s,\widehat{\boldsymbol{\beta}})}(\widehat{\boldsymbol{\beta}}-\boldsymbol{\beta}^0) + o_p\left(1/\sqrt{n}\right)$$

$$= -\left[\int_{a_T}^t \overline{\boldsymbol{x}}_{\boldsymbol{a}}(s,\boldsymbol{\beta}^0)d\widehat{\Lambda}_0(s;\boldsymbol{\beta}^0,\boldsymbol{a})\right]^\intercal \frac{1}{n}\sum_{i=1}^n L_i^{\boldsymbol{\beta}} + o_p\left(1/\sqrt{n}\right).$$

Through the arguments that follow, we can further show that $\widehat{\Lambda}_0(s;\boldsymbol{\beta}^0,\boldsymbol{a})$ converges to $\Lambda_0(t)$ uniformly in $t \in [a_T,\tau]$, so $\int_{a_T}^t \overline{\boldsymbol{x}}_{\boldsymbol{a}}(s,\boldsymbol{\beta}^0)d\widehat{\Lambda}_0(s;\boldsymbol{\beta}^0,\boldsymbol{a}) = \int_{a_T}^t \boldsymbol{e}_{\boldsymbol{a}}(s,\boldsymbol{\beta}^0)d\Lambda_0(s) + o_p(1)$.

Next, it is straightforward to verify that $S_2 = \Lambda_0(t)$. For S_3 we have

$$S_3 = \frac{1}{n}\sum_{i=1}^n \int_{a_T}^t \frac{\left[\widehat{a}(T_i)^{-1} - a(T_i)^{-1}\right]dM_i(s)}{\boldsymbol{s}_{\boldsymbol{a}}^{(0)}(s,\boldsymbol{\beta}^0)} + o_p\left(1/\sqrt{n}\right)$$

$$= \frac{1}{n^2} \sum_{i=1}^{n} \sum_{j=1}^{n} L_j(T_i) \int_{a_T}^{t} \frac{dM_i(s)}{\boldsymbol{s}_{\boldsymbol{a}}^{(0)}(s, \boldsymbol{\beta}^0)} + o_p\left(1/\sqrt{n}\right)$$

$$= \frac{1}{2n^2} \sum_{i \neq j} \left[L_j(T_i) \int_{a_T}^{t} \frac{dM_i(s)}{\boldsymbol{s}_{\boldsymbol{a}}^{(0)}(s, \boldsymbol{\beta}^0)} + L_i(T_j) \int_{a_T}^{t} \frac{dM_j(s)}{\boldsymbol{s}_{\boldsymbol{a}}^{(0)}(s, \boldsymbol{\beta}^0)} \right] + o_p\left(1/\sqrt{n}\right),$$

which is a stochastic process of mean-zero U-statistics indexed by t. Then, noting that $L_j(t)$ has uniformly mean zero, by the theory of U-processes (Nolan and Pollard, 1988) we have

$$S_3 = \frac{1}{n} \sum_{i=1}^{n} \mathbb{E}^* \left[L_i(T_0) \int_{a_T}^{t} \frac{dM_0(s)}{\boldsymbol{s}_{\boldsymbol{a}}^{(0)}(s, \boldsymbol{\beta}^0)} \Big| \boldsymbol{D}_i \right] + o_p\left(1/\sqrt{n}\right).$$

Finally, for S_4 we have

$$S_4 = \frac{1}{n} \sum_{i=1}^{n} \int_{a_T}^{t} \frac{a(T_i)^{-1} dM_i(s)}{\boldsymbol{s}_{\boldsymbol{a}}^{(0)}(s, \boldsymbol{\beta}^0)} + o_p\left(1/\sqrt{n}\right).$$

□

S5 Additional simulation results

S5.1 NPMLE

We conducted additional simulations to assess the accuracy of the proposed standard errors for the NPMLE. The event times were distributed as Uniform$(0.1, 1.1)$, while the truncation times were $U_i \sim$ Uniform$(0, 1)$ and $V_i = U_i + D_i$, with $D_i \sim$ Uniform$(0.1, 0.6)$. All times were rounded to three decimal places, resulting in an average of 110 tied event times out of the total sample size of 500. Overall truncation probabilities were 40% on the left and 29% on the right. We considered the nonparametric maximum likelihood estimates of the weighting process $a(t)$, event time distribution function $F(t)$, and event time hazard function $\Lambda(t)$.

Figure S1 contains the results for these simulations. All estimators are approximately unbiased. The proposed standard errors are generally well-calibrated, although they can be slightly conservative, particularly for the event time cdf.

Then, we evaluated testing procedures for assessing ignorable sampling bias using the proposed standard errors. The first simulation settings are a subset of those considered by de Uña-Álvarez (2023), who proposed using the test statistic $\sup|\widehat{F}(t) - \widehat{F^*}(t)|$ but relied on bootstrap resampling to obtain p-values. The event times had a Uniform(0,1) distribution, while the truncation times were generated as $U_i = (1+\zeta)Z_i^{\rho} - \zeta$ and $V_i = U_i + \zeta$, for Uniform(0,1) random variables Z_i. For $\zeta \in \{0.5, 1\}$, higher values shifted the sampling probability $\pi(t)$ away from zero. The parameter $\rho \in \{1, 2, 6\}$ reflected no, small, and large departures from the null hypothesis, respectively. Note that, in all cases, the sampling probability is a monotone function. The sample size was 200 and we conducted 1000 simulations for each setting. We evaluated the two supremum tests discussed in the main text, one based on the cdf and the other based on the weighting process $a(t)$. We used our closed-form influence function estimates to sample from the estimated asymptotic

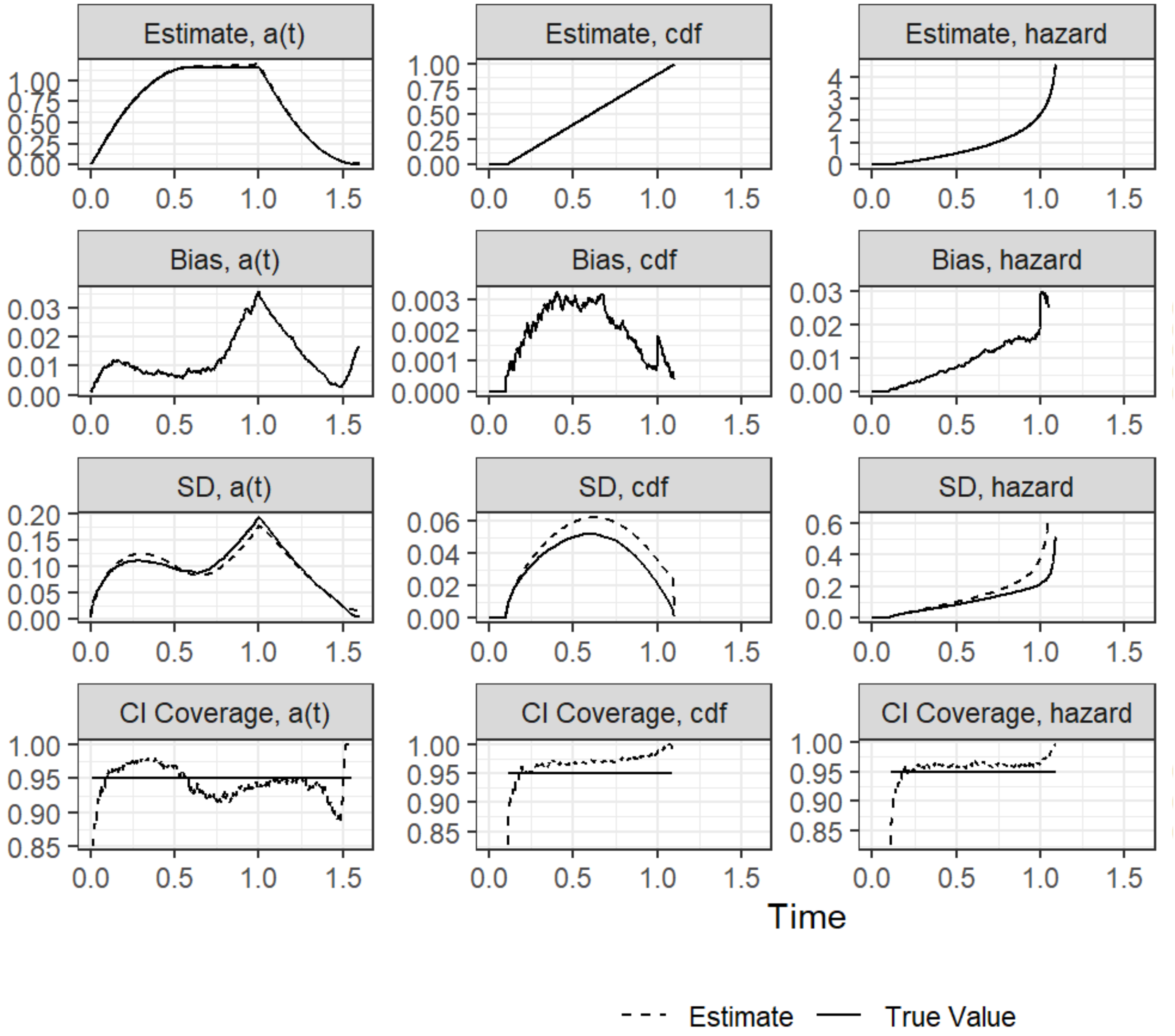

Figure S1: Simulation results for nonparametric analysis of doubly truncated data. From left to right, the panels pertain to the NPMLE for the weighting process $a(t)$, the event time distribution $F(t)$, and the event time cumulative hazard $\Lambda(t)$. Estimates are plotted in dashed lines, while the true values are solid lines. Coverage probability is plotted for pointwise linear confidence intervals.

Table S1: Simulation results for nonparametric ignorable sampling bias testing at significance level 0.05.

ζ	ρ	Test	Median Test Statistic	Median Critical Value	Rejection Rate
0.5	1	$\sup \lvert \widehat{F}(t) - \widehat{F^*}(t) \rvert$	0.055	0.055	0.070
		$\sup \lvert \widehat{a}(t) - 1 \rvert$	0.399	0.784	0.012
	2	$\sup \lvert \widehat{F}(t) - \widehat{F^*}(t) \rvert$	0.118	0.118	0.521
		$\sup \lvert \widehat{a}(t) - 1 \rvert$	1.104	1.225	0.257
	6	$\sup \lvert \widehat{F}(t) - \widehat{F^*}(t) \rvert$	0.208	0.208	0.909
		$\sup \lvert \widehat{a}(t) - 1 \rvert$	3.211	2.857	0.604
1.0	1	$\sup \lvert \widehat{F}(t) - \widehat{F^*}(t) \rvert$	0.026	0.026	0.038
		$\sup \lvert \widehat{a}(t) - 1 \rvert$	0.196	0.365	0.029
	2	$\sup \lvert \widehat{F}(t) - \widehat{F^*}(t) \rvert$	0.088	0.088	0.824
		$\sup \lvert \widehat{a}(t) - 1 \rvert$	0.709	0.643	0.533
	6	$\sup \lvert \widehat{F}(t) - \widehat{F^*}(t) \rvert$	0.164	0.164	0.998
		$\sup \lvert \widehat{a}(t) - 1 \rvert$	2.060	1.630	0.695

null distributions without re-fitting the NPMLE, similar to our quasi-independent truncation diagnostic. In each test, we sampled 2000 realizations of the associated Gaussian process under the null hypothesis, computed the supremum for each one, and estimated the p-value from the fraction of simulated supremums that were greater than the observed test statistic. Each realization was a weighted average of the n subject-specific influence function estimates, with weights generated from the Exponential(1) distribution and divided by n (see Section S2 for more details).

The simulation results for the ignorable sampling bias tests are summarized in Table S1. Under the null hypothesis ($\rho = 1$), both tests control the Type I error rate at the nominal level 0.05. The test based on the weighting process $a(t)$ tends to be more conservative than the test based on the event time cdf. Both tests become more powerful as the sampling probability shifts away from zero. This is expected since near-violations of the positivity assumption will generally inflate the variance of the NPMLE. Using our closed-form standard errors instead of bootstrap resampling generally increases the power of the cdf-based test. For example, at $(\zeta, \rho) = (0.5, 2)$ our rejection rate is 0.52, compared to 0.33 in Table 1 of de Uña-Álvarez (2023). Similarly, at $(\zeta, \rho) = (0.5, 6)$ we have 0.91 compared to 0.77 for bootstrap resampling.

Finally, Table S2 contains a summary of the two ignorability tests when applied to the simulation setting of Figure S1. Here the test statistic based on $a(t)$ has drastically higher power compared to the cdf test. Therefore neither test completely dominates the other in all situations. The empirical results presented here suggest that the deciding factor could be related to whether $a(t)$ is a monotone function of time within the support of the event times, but further theoretical investigation is needed to confirm this conjecture.

Table S2: Simulation results for nonparametric ignorable sampling bias testing in the setting of Figure S1, at level 0.05.

Test	Median Test Statistic	Median Critical Value	Rejection Rate
$\sup \lvert \widehat{F}(t) - \widehat{F^*}(t) \rvert$	0.087	0.114	0.148
$\sup \lvert \widehat{a}(t) - 1 \rvert$	0.656	0.389	0.986

S5.2 Quasi-independent truncation diagnostic

In order to evaluate the performance of the proposed diagnostic test for quasi-independent truncation, we conducted simulations under dependent truncation. There were two covariates X_1 and X_2, which were independent discrete random variables with $\mathbb{P}(X_j = k) = 1/S$ for $j = 1, 2$ and $k = 1, \ldots, S$, where S was the total number of strata. The event time was generated from a Cox model $\lambda(t|\boldsymbol{x}) = \exp(\mathbf{1}(\boldsymbol{x}_1 = 1) + \mathbf{1}(\boldsymbol{x}_2 = 1))\lambda_0(t)$ with a beta(2, 1.5)$\times 0.4 + 0.1$ baseline hazard. The left truncation time U followed a beta(1, 3)$\times 0.5$ distribution, while the right truncation time was $V = U + 0.1 +$ beta(2, 10) $\times\, \Delta^{\mathbf{1}(X_1=1)}$ for a chosen constant $\Delta \in [1, 2]$. For $\Delta > 1$, the right truncation time distribution changed scale depending on the covariate value X_1, thus the magnitude of Δ determined the degree of violation of the quasi-independent truncation assumption. We generated 1000 simulations at various values of Δ and tracked the rejection rate for the null hypothesis that the quasi-independent truncation assumption holds. The diagnostic test was stratified by X_1 and p-values were computed by sampling from the estimated asymptotic null distribution as described in Section S2.

The results are plotted in Figure S2 for sample size $n \in \{200, 400\}$, total number of strata $S \in \{2, 3, 4, 5\}$, and significance level 0.05. Under quasi-independent truncation, where $\sup |\pi(t|s) - \pi(t)| = 0$, the diagnostic test has a Type I error rate approximately equal to the chosen significance level, though it can be slightly conservative when there are many strata. As the degree of dependent truncation, as measured by $\sup |\pi(t|s) - \pi(t)|$, increases, the rejection rate rises towards one and the test has high power even in small samples, again as long as there are not too many strata. Each additional strata can reduce the test's power by half for small effect sizes, although the reduction is less severe for large effects. Thus, when applying this diagnostic test in practice, one should try to limit the total number of strata to 2-4 and also take into account the magnitude of the estimated effect size $\sup |\widehat{\pi}(t|s) - \widehat{\pi}(t)|$ when determining whether quasi-independent truncation is plausible. Towards this end, in the main text we propose a graphical diagnostic that can be overlaid with a uniform confidence band for $\sup |\widehat{\pi}(t|s) - \widehat{\pi}(t)|$.

S5.3 Additional simulations for robust IPW Cox regression

S5.3.1 Well-behaved data

We ran further simulations to evaluate the various IPW estimators and the proposed standard errors. To vary the truncation rate, we generated truncation times $U \sim \text{beta}(4, 8) \times 0.5 + 0.1 - k$ and $V = U + \text{beta}(6, 30) + 0.05 + 2k$ for $k \in \{0, 0.05, 0.1\}$. Increasing the value of k increases the separation between U and V, decreasing the probability of truncation. The

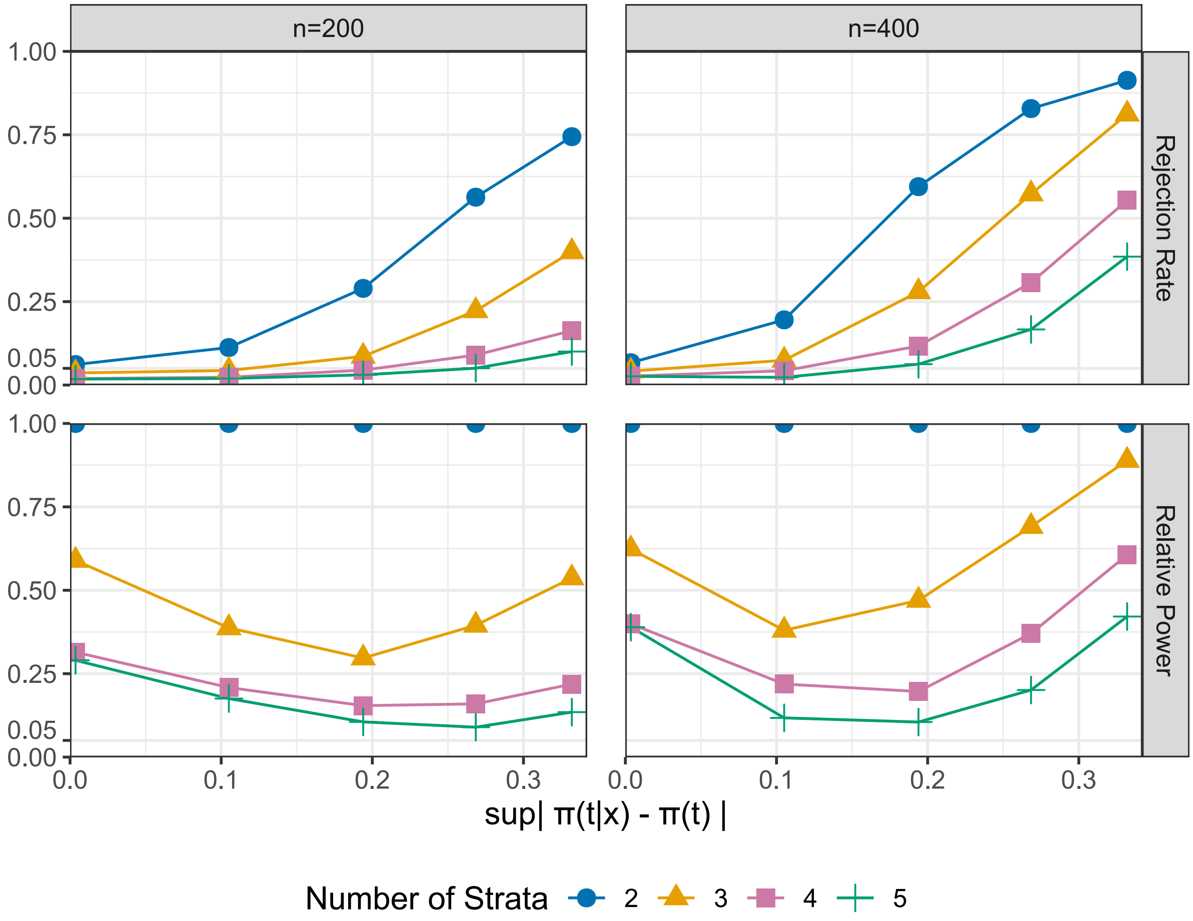

Figure S2: Simulation results for the quasi-independent truncation diagnostic test at various sample sizes and numbers of strata. Within each simulation, the p-value for the diagnostic test was computed by generating 2000 samples from the estimated asymptotic null distribution of the test statistic. Rejection rate was the fraction of simulations where the test rejected the null hypothesis at significance level 0.05. Relative power was computed by dividing the rejection rate from the test with two strata.

event times followed a Cox model with $\text{beta}(2, 1.1) \times 0.4 + 0.2$ baseline hazard, independent Bernoulli(0.5) and Uniform(0,1) covariates, and $\boldsymbol{\beta}^0 = (1, 1)^\mathsf{T}$. In order to further stabilize the estimators and their standard errors in the presence of large inverse probability weights, we artificially censored 2.5% of the data from each tail of the observed event times. This is an alternative to artificially truncating large estimated weights, with the advantage of not introducing any bias in the estimates. The results are summarized in Table S3. Here all IPW estimators are unbiased, and their confidence intervals have the correct 95% coverage probability when using the proposed adjusted standard errors (adj. or robust). As expected, the unadjusted sandwich standard errors (unadj.) tend to underestimate the true variability of the IPW estimators, especially for small samples or heavy truncation. On the other hand, including $\boldsymbol{U}_{w3i}$ (robust vs adj.) does not have a tangible impact on the accuracy of the standard errors because the fitted model is correctly specified. In general, all IPW estimators have roughly the same performance.

S5.3.2 Contaminated data

We also ran the contaminated data simulations of the main text (see Table 1) at different sample sizes. Table S4 contains the results at $n = 300$, while Table S5 contains the results at $n = 700$. We observe the same trends as in Table 1. In particular, the proposed estimators achieve much lower MSE than the existing estimators under data contamination.

S5.4 Non-proportional hazards

Suppose that the true log hazard ratio, $\boldsymbol{\beta}^0(t)$, varies over time. Directly estimating $\boldsymbol{\beta}^0(t)$ to perform inference on a covariate effect may be cumbersome, and the results are difficult to interpret. But following Xu and O'Quigley (2000) one can show that an IPW Cox regression estimator $\widehat{\boldsymbol{\beta}}_w$ provides a meaningful summary of the regression effect by averaging it over time, with limiting value

$$\boldsymbol{\beta}^*_w \approx \frac{\mathbb{E}[w(T)\boldsymbol{\beta}^0(T)]}{\mathbb{E}[w(T)]}.$$

Thus weighting this average by the survival function $w(t) = S(t)$, i.e. the proportion of pre-truncation individuals at-risk at a given time, has a clear interpretation in this context, because more importance is assigned to event times where many people are affected.

In untruncated Cox regression, Schemper et al. (2009) also advocated for survival function weighting under non-proportional hazards. They showed that, for a binary covariate $\boldsymbol{x} \in \{0, 1\}$ and concordance probability defined as $\mathbb{P}(T_1 < T_2 | \boldsymbol{x}_1 = 1, \boldsymbol{x}_2 = 0)$, survival function weighting leads to "hazard ratio" estimates $\exp(\widehat{\boldsymbol{\beta}}_w)$ that closely approximate the odds of concordance, which has a clear interpretation even under non-proportional hazards. The same was not true when using the standard partial likelihood estimator, a choice which is analogous to using an IPW estimator with $\widehat{w}(t) = 1$ for doubly truncated data. We present similar findings for IPW estimators in the simulation results below, which support the use of survival function weighting under possibly non-proportional hazards.

We simulated two examples of non-proportional hazards for a single binary covariate $X \sim \text{Bernoulli}(0.5)$. The truncation times were generated as in Table 2 in the main text. In

Table S3: Simulation results for regression coefficient estimation at varying levels of double truncation. Includes estimators for $\boldsymbol{\beta}_1^0 = 1$ using the standard unadjusted (UW) and IPW partial likelihood (W-1), as well as time-varying weights based on the survival function $1 - \widehat{F}(t)$ (W-surv), Fleming-Harrington weights $\widehat{F}(t)^{1/2}[1 - \widehat{F}(t)]^{1/2}$ (W-fh), and their stabilized weighting counterparts $\widehat{a}(t)$ (W-a), $\widehat{a}(t)[1 - \widehat{F}(t)]$ (W-asurv), and $\widehat{a}(t)\widehat{F}(t)^{1/2}[1 - \widehat{F}(t)]^{1/2}$ (W-afh). 95% confidence intervals (CI) are obtained from a normal approximation and either unadjusted sandwich standard errors (unadj.) or the proposed standard errors that account for NPMLE variability (adj./robust).

					Standard Error			CI Coverage		
						Proposed			Proposed	
n	Estimator	Bias	MSE	SD	unadj.	adj.	robust	unadj.	adj.	robust
				Light Truncation: 6% left, 12% right, 19% overall						
200	W-1	0.01	0.039	0.196	0.180	0.182		0.938	0.939	
	W-surv	0.01	0.039	0.198	0.190	0.191	0.192	0.938	0.942	0.943
	W-fh	0.01	0.038	0.194	0.182	0.183	0.185	0.934	0.937	0.939
	W-a	0.01	0.037	0.192	0.181	0.182	0.182	0.941	0.944	0.944
	W-asurv	0.01	0.038	0.195	0.187	0.189	0.189	0.940	0.947	0.946
	W-afh	0.01	0.037	0.193	0.183	0.185	0.186	0.935	0.938	0.941
	UW	-0.16	0.051	0.156	0.152			0.781		
500	W-1	0.00	0.014	0.120	0.117	0.117		0.940	0.939	
	W-surv	0.00	0.014	0.118	0.120	0.121	0.121	0.953	0.955	0.956
	W-fh	0.00	0.014	0.119	0.117	0.118	0.118	0.939	0.942	0.944
	W-a	0.00	0.014	0.117	0.116	0.117	0.117	0.942	0.943	0.944
	W-asurv	0.00	0.014	0.117	0.119	0.120	0.120	0.955	0.955	0.955
	W-afh	0.00	0.014	0.118	0.117	0.118	0.119	0.943	0.946	0.947
	UW	-0.17	0.039	0.094	0.096			0.581		
				Moderate Truncation: 15% left, 22% right, 37% overall						
200	W-1	0.01	0.047	0.216	0.198	0.203		0.934	0.941	
	W-surv	0.01	0.045	0.211	0.204	0.209	0.210	0.944	0.950	0.950
	W-fh	0.01	0.047	0.216	0.198	0.203	0.205	0.933	0.941	0.941
	W-a	0.01	0.045	0.212	0.197	0.202	0.201	0.936	0.939	0.940
	W-asurv	0.01	0.043	0.208	0.198	0.203	0.204	0.935	0.942	0.943
	W-afh	0.01	0.046	0.215	0.199	0.204	0.205	0.933	0.940	0.940
	UW	-0.24	0.082	0.157	0.150			0.618		
500	W-1	0.01	0.020	0.143	0.129	0.132		0.940	0.941	
	W-surv	0.00	0.019	0.138	0.129	0.132	0.132	0.946	0.952	0.953
	W-fh	0.01	0.020	0.141	0.128	0.131	0.132	0.934	0.940	0.941
	W-a	0.00	0.019	0.138	0.127	0.130	0.129	0.940	0.944	0.944
	W-asurv	0.00	0.018	0.135	0.126	0.129	0.129	0.944	0.949	0.949
	W-afh	0.00	0.019	0.138	0.128	0.131	0.131	0.935	0.942	0.942
	UW	-0.24	0.068	0.099	0.095			0.299		
				Heavy Truncation: 28% left, 35% right, 63% overall						
200	W-1	0.01	0.064	0.254	0.211	0.227		0.902	0.925	
	W-surv	0.00	0.060	0.244	0.217	0.232	0.233	0.929	0.949	0.950
	W-fh	0.01	0.063	0.251	0.210	0.225	0.229	0.905	0.924	0.927
	W-a	0.01	0.059	0.242	0.209	0.224	0.223	0.916	0.934	0.930
	W-asurv	0.00	0.054	0.233	0.207	0.221	0.222	0.923	0.943	0.942
	W-afh	0.01	0.059	0.243	0.210	0.225	0.226	0.914	0.928	0.928
	UW	-0.29	0.109	0.160	0.149			0.503		
500	W-1	0.00	0.025	0.157	0.140	0.150		0.924	0.938	
	W-surv	0.00	0.023	0.152	0.138	0.147	0.148	0.938	0.953	0.954
	W-fh	0.00	0.024	0.153	0.138	0.148	0.148	0.925	0.946	0.948
	W-a	0.00	0.022	0.150	0.137	0.146	0.146	0.936	0.951	0.951
	W-asurv	0.00	0.021	0.146	0.132	0.142	0.142	0.935	0.956	0.956
	W-afh	0.00	0.022	0.150	0.137	0.146	0.146	0.933	0.950	0.951
	UW	-0.29	0.092	0.095	0.094			0.153		

Table S4: Simulation results for regression coefficient estimation with contaminated data at $n = 300$. The truncation rates were 0.15 (left), 0.21 (right), 0.36 (overall). Includes estimators for $\boldsymbol{\beta}_1^0 = 1$ using the standard unadjusted (UW) and IPW partial likelihood (W-1), as well as time-varying weights based on the survival function $1-\widehat{F}(t)$ (W-surv), general Fleming-Harrington weights $\widehat{F}(t)^r[1-\widehat{F}(t)]^s$ (W-fhrs), and their stabilized weighting counterparts $\widehat{a}(t)$ (W-a), $\widehat{a}(t)[1-\widehat{F}(t)]$ (W-asurv), and $\widehat{a}(t)\widehat{F}(t)^r[1-\widehat{F}(t)]^s$ (W-afhrs). 95% confidence intervals (CI) are obtained from a normal approximation and either unadjusted sandwich standard errors (unadj.) or the proposed standard errors that account for the NPMLE variability (adj. and robust).

				Standard Error			CI Coverage		
					Proposed			Proposed	
Estimator	Bias (%)	MSE	SD	unadj.	adj.	robust	unadj.	adj.	robust
				No data contamination					
W-1[a]	1.18	0.024	0.156	0.141	0.145		0.919	0.931	
W-surv[d]	0.78	0.025	0.158	0.150	0.153	0.154	0.937	0.942	0.941
W-fh02[c]	0.94	0.031	0.176	0.169	0.172	0.172	0.934	0.943	0.943
W-fh11[c]	0.80	0.026	0.161	0.148	0.152	0.153	0.926	0.933	0.936
W-a[b]	1.03	0.024	0.153	0.141	0.145	0.145	0.926	0.934	0.932
W-asurv[d]	0.76	0.025	0.157	0.149	0.152	0.153	0.936	0.942	0.943
W-afh02[c]	0.89	0.030	0.172	0.165	0.168	0.168	0.935	0.942	0.942
W-afh11[c]	0.74	0.026	0.160	0.149	0.153	0.154	0.929	0.932	0.933
MLE	1.93	0.031	0.176						
UW	-13.17	0.034	0.129	0.124			0.801		
				Contamination probability: 1%					
W-1[a]	-10.13	0.048	0.195	0.146	0.150		0.838	0.845	
W-surv[d]	-5.50	0.030	0.164	0.151	0.154	0.154	0.921	0.926	0.926
W-fh02[c]	-4.17	0.033	0.177	0.169	0.172	0.172	0.937	0.943	0.941
W-fh11[c]	-7.68	0.038	0.178	0.153	0.156	0.157	0.886	0.894	0.897
W-a[b]	-8.55	0.040	0.180	0.146	0.148	0.148	0.867	0.873	0.872
W-asurv[d]	-5.15	0.029	0.163	0.151	0.153	0.154	0.921	0.929	0.931
W-afh02[c]	-4.08	0.032	0.175	0.166	0.169	0.169	0.937	0.942	0.941
W-afh11[c]	-6.94	0.035	0.175	0.153	0.156	0.157	0.895	0.901	0.903
MLE	-7.60	0.044	0.195						
UW	-20.31	0.062	0.145	0.127			0.617		
				Contamination probability: 3%					
W-1[a]	-26.80	0.112	0.199	0.158	0.159		0.577	0.582	
W-surv[d]	-14.58	0.046	0.159	0.153	0.155	0.155	0.830	0.835	0.838
W-fh02[c]	-10.62	0.041	0.172	0.169	0.171	0.171	0.903	0.908	0.909
W-fh11[c]	-21.55	0.077	0.174	0.160	0.161	0.162	0.708	0.709	0.714
W-a[b]	-22.97	0.085	0.179	0.154	0.155	0.156	0.654	0.659	0.660
W-asurv[d]	-13.82	0.044	0.158	0.152	0.154	0.154	0.845	0.847	0.850
W-afh02[c]	-10.52	0.040	0.169	0.166	0.168	0.168	0.896	0.900	0.900
W-afh11[c]	-19.73	0.067	0.169	0.159	0.160	0.161	0.739	0.740	0.745
MLE	-20.12	0.075	0.186						
UW	-30.93	0.115	0.140	0.129			0.352		

[a] Rennert and Xie (2018).
[b] Mandel et al. (2018).
[c] Weights motivated by the $G^{r,s}$ tests of Fleming and Harrington (2013).
[d] Proposed estimators.

Table S5: Simulation results for regression coefficient estimation with contaminated data at $n = 700$. The truncation rates were 0.15 (left), 0.21 (right), 0.36 (overall). Includes estimators for $\boldsymbol{\beta}_1^0 = 1$ using the standard unadjusted (UW) and IPW partial likelihood (W-1), as well as time-varying weights based on the survival function $1-\widehat{F}(t)$ (W-surv), general Fleming-Harrington weights $\widehat{F}(t)^r[1-\widehat{F}(t)]^s$ (W-fhrs), and their stabilized weighting counterparts $\widehat{a}(t)$ (W-a), $\widehat{a}(t)[1-\widehat{F}(t)]$ (W-asurv), and $\widehat{a}(t)\widehat{F}(t)^r[1-\widehat{F}(t)]^s$ (W-afhrs). 95% confidence intervals (CI) are obtained from a normal approximation and either unadjusted sandwich standard errors (unadj.) or the proposed standard errors that account for the NPMLE variability (adj. and robust).

				Standard Error			CI Coverage		
					Proposed			Proposed	
Estimator	Bias (%)	MSE	SD	unadj.	adj.	robust	unadj.	adj.	robust
				No data contamination					
W-1[a]	0.41	0.010	0.098	0.093	0.095		0.946	0.950	
W-surv[d]	0.15	0.010	0.102	0.098	0.100	0.100	0.952	0.955	0.955
W-fh02[c]	0.04	0.013	0.116	0.110	0.113	0.113	0.944	0.951	0.950
W-fh11[c]	0.49	0.010	0.102	0.097	0.099	0.100	0.944	0.948	0.949
W-a[b]	0.40	0.009	0.097	0.093	0.095	0.095	0.947	0.952	0.952
W-asurv[d]	0.20	0.010	0.102	0.097	0.099	0.100	0.953	0.956	0.956
W-afh02[c]	0.09	0.013	0.114	0.108	0.110	0.110	0.944	0.949	0.950
W-afh11[c]	0.49	0.010	0.102	0.098	0.100	0.100	0.941	0.948	0.948
MLE	0.59	0.012	0.110						
UW	-13.51	0.025	0.083	0.081			0.600		
				Contamination probability: 1%					
W-1[a]	-13.41	0.036	0.135	0.108	0.108		0.742	0.745	
W-surv[d]	-5.99	0.014	0.102	0.100	0.102	0.102	0.906	0.910	0.912
W-fh02[c]	-3.89	0.014	0.110	0.111	0.113	0.112	0.945	0.948	0.947
W-fh11[c]	-9.80	0.022	0.113	0.105	0.106	0.106	0.832	0.834	0.839
W-a[b]	-10.95	0.026	0.119	0.103	0.104	0.104	0.799	0.804	0.805
W-asurv[d]	-5.62	0.013	0.101	0.099	0.101	0.101	0.911	0.915	0.916
W-afh02[c]	-3.87	0.013	0.108	0.109	0.110	0.110	0.940	0.942	0.941
W-afh11[c]	-8.84	0.020	0.110	0.104	0.105	0.105	0.857	0.861	0.864
MLE	-9.86	0.025	0.122						
UW	-21.87	0.056	0.093	0.086			0.314		
				Contamination probability: 3%					
W-1[a]	-29.86	0.107	0.133	0.113	0.114		0.295	0.297	
W-surv[d]	-14.87	0.033	0.106	0.101	0.102	0.102	0.683	0.688	0.688
W-fh02[c]	-10.47	0.024	0.114	0.111	0.112	0.112	0.830	0.833	0.833
W-fh11[c]	-22.83	0.066	0.117	0.109	0.109	0.110	0.436	0.438	0.443
W-a[b]	-24.79	0.076	0.119	0.107	0.107	0.108	0.373	0.380	0.380
W-asurv[d]	-14.05	0.031	0.106	0.101	0.102	0.102	0.696	0.701	0.700
W-afh02[c]	-10.43	0.024	0.113	0.109	0.110	0.110	0.828	0.828	0.829
W-afh11[c]	-20.72	0.056	0.114	0.107	0.108	0.108	0.502	0.503	0.506
MLE	-22.34	0.065	0.124						
UW	-32.45	0.114	0.094	0.086			0.062		

[a] Rennert and Xie (2018).
[b] Mandel et al. (2018).
[c] Weights motivated by the $G^{r,s}$ tests of Fleming and Harrington (2013).
[d] Proposed estimators.

Table S6: Simulation results for regression coefficient estimation under non-proportional hazards. Includes estimators using the standard IPW partial likelihood (W-1), as well as time-varying weights based on the survival function $1-\widehat{F}(t)$ (W-surv), Fleming-Harrington weights $[1-\widehat{F}(t)]^{1/2}\widehat{F}(t)^{1/2}$ (W-fh), and their stabilized weighting counterparts $\widehat{a}(t)$ (W-a), $\widehat{a}(t)[1-\widehat{F}(t)]$ (W-asurv), and $\widehat{a}(t)[1-\widehat{F}(t)]^{1/2}\widehat{F}(t)^{1/2}$ (W-afh). Here UT refers to the unweighted estimator fit on untruncated data. 95% confidence intervals (CI) are obtained from a normal approximation and either unadjusted sandwich standard errors (unadj.) or the proposed standard errors that account for the NPMLE variability (adj. and robust). Note only (robust) are consistent under model misspecification.

			Standard Error			CI Coverage		
				Proposed			Proposed	
Estimator	$\widehat{\beta}$	SD	unadj.	adj.	robust	unadj.	adj.	robust
Converging hazards (log-odds of concordance=0.69)								
W-1	0.45	0.134	0.126	0.127		0.939	0.946	
W-surv	0.68	0.124	0.120	0.121	0.122	0.943	0.943	0.946
W-fh	0.39	0.133	0.127	0.127	0.130	0.947	0.948	0.950
W-a	0.47	0.126	0.122	0.123	0.122	0.948	0.949	0.950
W-asurv	0.64	0.123	0.121	0.121	0.121	0.945	0.947	0.949
W-afh	0.41	0.130	0.126	0.126	0.127	0.953	0.953	0.953
UT	0.45	0.095	0.093			0.943		
Crossing hazards (log-odds of concordance=0)								
W-1	0.43	0.126	0.114	0.124		0.927	0.949	
W-surv	0.00	0.147	0.118	0.127	0.135	0.889	0.918	0.943
W-fh	0.61	0.141	0.120	0.127	0.135	0.901	0.923	0.948
W-a	0.36	0.119	0.115	0.124	0.118	0.950	0.965	0.954
W-asurv	-0.01	0.136	0.119	0.127	0.128	0.919	0.941	0.942
W-afh	0.53	0.134	0.121	0.127	0.130	0.924	0.944	0.954
UT	0.43	0.091	0.095			0.960		

both examples the event times for the group with $X=0$ had a Uniform$(0.1, 0.5)$ distribution, while for $X=1$ the distribution was either beta$(0.5, 1) \times 0.4 + 0.1$ (converging hazards) or beta$(5, 5) \times 0.4 + 0.1$ (crossing hazards). Table S6 summarizes the results from these simulations at $n = 500$, where the standard partial likelihood estimator fit on untruncated data (UT) is also provided for reference. Only W-surv provides estimates closely approximating the odds of concordance, which still has a clear interpretation under non-proportional hazards. This is in-line with the results of Schemper et al. (2009) for untruncated data. On the other hand, the standard IPW maximum partial likelihood estimator (W-1) is equivalent to unweighted Cox regression for untruncated data (UT) in terms of their limiting value $\boldsymbol{\beta}_w^*$. For crossing hazards, only the proposed standard errors that include $\boldsymbol{U}_{w3i}$ (robust) are accurate when using time-varying weights, since they are robust to model misspecification.

References

de Uña-Álvarez, J. and Keilegom, I. V. (2021). Efron–Petrosian integrals for doubly truncated data with covariates: An asymptotic analysis. *Bernoulli*, 27(1):249 – 273.

de Uña-Álvarez, J. (2023). Testing for an ignorable sampling bias under random double truncation. *Statistics in Medicine*, 42(20):3732–3744.

Fleming, T. R. and Harrington, D. P. (2013). *Counting processes and survival analysis*, volume 625. John Wiley & Sons.

Fleming, T. R., Harrington, D. P., and O'sullivan, M. (1987). Supremum versions of the log-rank and generalized wilcoxon statistics. *Journal of the American Statistical Association*, 82(397):312–320.

Mandel, M., de Uña-Álvarez, J., Simon, D. K., and Betensky, R. A. (2018). Inverse probability weighted Cox regression for doubly truncated data. *Biometrics*, 74(2):481–487.

Nolan, D. and Pollard, D. (1988). Functional limit theorems for u-processes. *The Annals of Probability*, 16(3):1291–1298.

Rennert, L. and Xie, S. X. (2018). Cox regression model with doubly truncated data. *Biometrics*, 74(2):725–733.

Schemper, M., Wakounig, S., and Heinze, G. (2009). The estimation of average hazard ratios by weighted Cox regression. *Statistics in medicine*, 28(19):2473–2489.

Xu, R. and O'Quigley, J. (2000). Estimating average regression effect under non-proportional hazards. *Biostatistics*, 1(4):423–439.
